# Polymer Network Engineering of Hydrogel Bioinks for Skin Regeneration: From Molecular Design to Adaptive Biofabrication

**DOI:** 10.3390/polym18212738

**Published:** 2026-11-04

**Authors:** Xuanru Zhu, Jiaxian Zhang, Hongwei Liu, Yuesheng Huang

**Affiliations:** 1 The First Clinical Medical College & The First Affiliated Hospital of Jinan University, Guangzhou 510632, China; 2 Institute of Wound Repair and Regeneration Medicine, School of Medicine, Southern University of Science and Technology, Shenzhen 518055, China

**Keywords:** hydrogel bioinks, skin tissue engineering, polymer engineering, wound healing, biofabrication, bibliometric analysis

## Abstract

Three-dimensional (3D) bioprinting offers substantial potential for reconstructing the structural and biological complexity of skin, yet its regenerative performance and clinical translation are constrained by the challenge of concurrently realizing printability, structural stability, and tissue remodeling with hydrogel bioinks. This review integrates bibliometric analysis with a polymer-centric assessment of functional hydrogel bioinks for skin tissue engineering and wound regeneration. Beyond polymer composition alone, we discuss how molecular features, crosslinking chemistry, and network architecture collectively determine bioink performance and regenerative function. Recent studies show that dynamic crosslinking, interpenetrating networks, and hybrid or nanocomposite designs can improve the balance between printability and post-print stability while also regulating transport, degradation, and cell–material interactions. Importantly, these material properties are not independent design targets; they are mechanistically linked to the ability of bioinks to modulate the wound microenvironment, support vascularization, and promote extracellular matrix reconstruction. Although emerging biofabrication strategies extend these functionalities, they impose stricter requirements for precise regulation of polymer rheology, crosslinking kinetics, and dynamic network adaptability. Despite rapid progress, translation to clinical use remains hindered by limited biological fidelity, poor reproducibility, and the lack of standardized manufacturing and regulatory criteria. We therefore propose that future bioink development should shift from empirical formulation toward predictive and adaptive polymer systems in which network design, biological function, and manufacturing requirements are considered simultaneously. This structure–property–function perspective provides a rational framework for developing next-generation polymer bioinks with improved regenerative efficacy and translational potential.

## 1. Introduction

Skin functions as the body’s primary barrier against environmental insults, playing an essential role in immune defense, thermoregulation, and sensory perception [1]. Extensive skin defects arising from severe burns, trauma, diabetes, or chronic ulcers present major clinical challenges, as they frequently lead to delayed healing, infection, excessive scarring, and incomplete functional recovery [2]. Existing treatments, including wound dressings, autografts, and engineered substitutes, have significantly improved wound management [3]. However, these approaches remain limited by donor tissue availability, immune compatibility, insufficient vascularization, and an inability to fully restore the structural and functional complexity of native skin [4]. These limitations have driven increasing interest in regenerative strategies capable of promoting functional tissue reconstruction. Skin tissue engineering has thus emerged as a promising approach that integrates biomaterials, cells, and biological cues to recreate a regenerative microenvironment [5].

Three-dimensional (3D) bioprinting has emerged as a powerful biofabrication technology for constructing biomimetic skin substitutes with a controlled architecture and spatial organization. Unlike conventional scaffold fabrication, bioprinting enables the precise deposition of biomaterials, living cells, and bioactive components into predefined structures, offering greater flexibility for recreating the structural and biological complexity of native skin [6]. The performance of this technology, however, relies heavily on the properties of the selected bioink. The term bioink generally refers to formulations containing cells or cell aggregates that are processed during bioprinting, either alone or in combination with biomaterials [7]. Among various bioink candidates, hydrogel-based bioinks have become the predominant choice for skin tissue engineering because their highly hydrated networks resemble the native extracellular matrix (ECM) and provide a favorable microenvironment for cell encapsulation, proliferation, and tissue remodeling. Beyond serving as passive cell carriers, hydrogel bioinks can be engineered to exhibit tunable rheological properties, controllable crosslinking kinetics, and programmable degradation, satisfying both the stringent requirements of printing and post-printing structural integrity [8]. Recent advances have further transformed these materials into functional platforms that can regulate the wound microenvironment and support tissue regeneration. These new properties have shifted the design focus from mere printability to integrating biological functionality with manufacturing performance, placing precise polymer engineering at the core of bioink development.

The performance of hydrogel bioinks is fundamentally determined by the intrinsic characteristics of their constituent polymers. Rational polymer design can regulate network formation and stability while also influencing degradation and biological interactions [9]. Natural polymers provide biological signals and ECM-like microenvironments, whereas synthetic polymers offer controllable mechanical properties and chemical versatility [10]. Hybrid systems and chemical modification further enable bioinks to acquire regenerative functions, including modulation of cellular responses and adaptation to pathological microenvironments. Developing next-generation bioinks therefore requires more than improving printability. And it also requires a clearer understanding of how polymer architecture determines material behavior and how these properties ultimately influence regenerative outcomes [11].

Several recent reviews have summarized the development of 3D bioprinting for skin regeneration. Most reviews have focused on printing technologies, commonly used biomaterials, or the reconstruction of specific skin functions. Other reviews have examined biomacromolecule-based bioinks, in situ bioprinting, or emerging technologies such as biosensor-integrated skin constructs. More recent work has provided broader overviews of bioinks and bioprinting strategies for structural and functional skin regeneration [12,13,14]. While these studies have substantially advanced the field, their discussions are generally focused on material categories, printing platforms, or regenerative applications, and relatively little attention has been paid to the polymer-network principles that bridge material structure with bioink performance and subsequent biological outcomes.

To address this gap, we conducted this review from a polymer-centered perspective on hydrogel bioinks for skin tissue engineering and wound regeneration. Accordingly, the scope of the review is restricted to hydrogel systems directly related to polymer network engineering and 3D bioprinting, excluding non-hydrogel bioinks and conventional scaffold-based approaches. We first used bibliometric analysis to characterize the evolution of the field and its emerging research directions. The subsequent review was organized around a structure–property–function framework that links polymer characteristics and network design to bioink behavior and regenerative outcomes. Rather than classifying materials solely by polymer type, we examine how network architecture governs material performance and how these properties subsequently influence biological function. Functional engineering and advanced biofabrication are therefore discussed within the same mechanistic framework. By integrating quantitative mapping of the literature with this polymer-centered analysis, we provide a distinct framework for the rational design and clinical translation of hydrogel bioinks.

## 2. Materials and Methods

### 2.1. Literature Search and Bibliometric Methodology

Relevant publications were retrieved from both the Web of Science Core Collection (WoSCC) and Scopus databases to improve our coverage of the literature and reduce database-specific bias. The literature search was completed on 8 September 2026, and no lower publication-year limit was applied. The search strategy was as follows:

(bioink OR “bio-ink” OR “3D bioprint*” OR “4D bioprint*” OR “bioprinted hydrogel*”) AND (hydrogel* OR “polymer hydrogel*” OR “polymeric hydrogel*” OR “natural polymer hydrogel*”) AND (“skin regeneration*” OR “wound healing*” OR “wound repair*” OR “skin tissue engineering*” OR “cutaneous regeneration*”).

Records retrieved from the two databases were merged, and duplicate publications were removed before bibliometric analysis. The resulting dataset was subsequently used to evaluate publication trends, collaboration networks, journal and co-citation patterns, and keyword evolution, thereby characterizing the development and emerging research directions of hydrogel bioinks for skin tissue engineering and wound regeneration.

### 2.2. Eligibility Criteria and Study Selection

**Figure 1:**
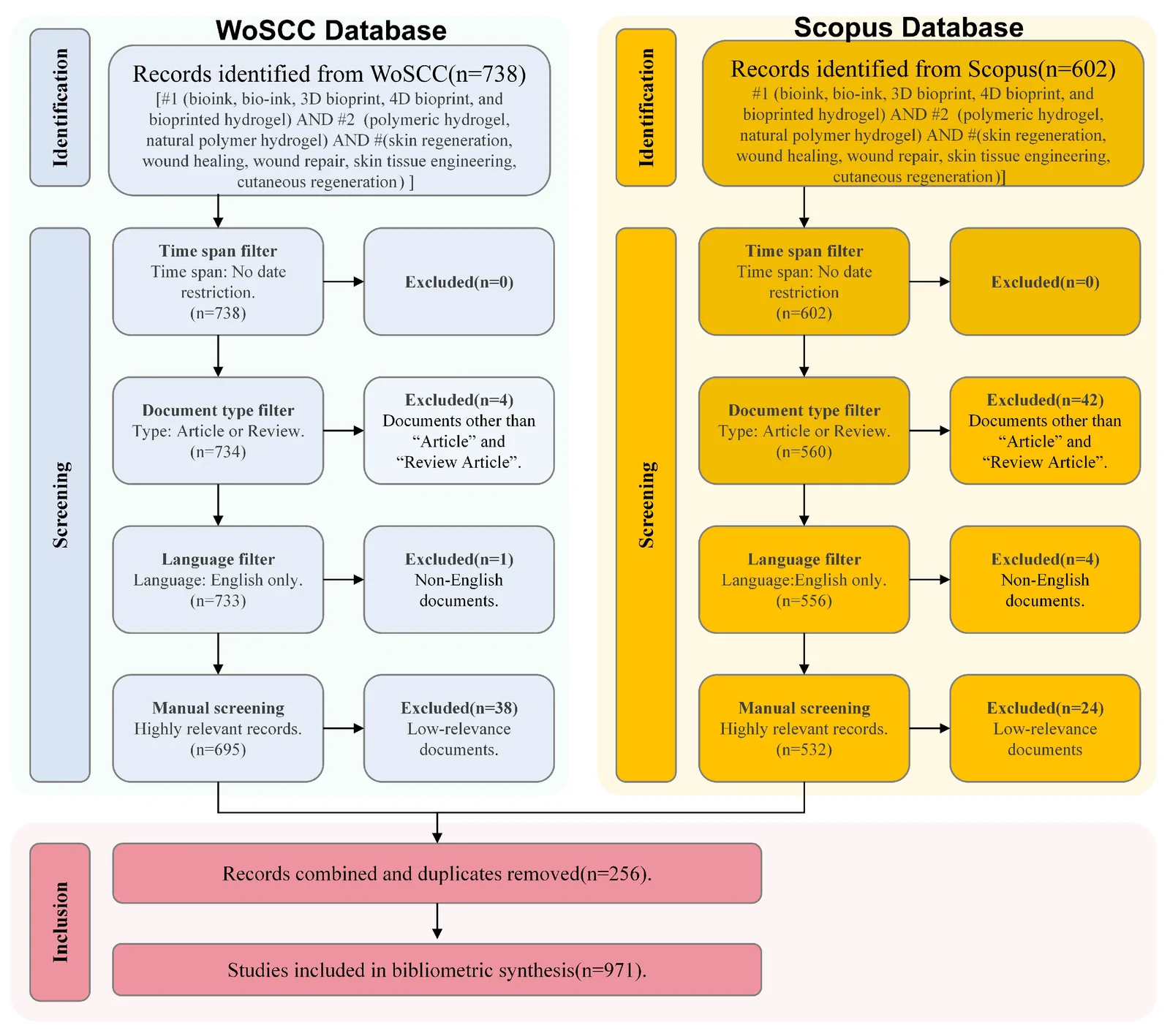
PRISMA 2020 flow diagram showing literature identification, screening, and inclusion for the bibliometric analysis. A total of 738 records were retrieved from the Web of Science Core Collection (WoSCC) and 602 from Scopus. After document-type, language, and relevance screening, 1227 records remained. Following the removal of 256 duplicate records, 971 unique publications were included in the bibliometric synthesis. No publication-year restriction was applied. # indicates the search level.

Studies were eligible if they addressed hydrogel-based bioinks or polymeric hydrogel systems for 3D/4D bioprinting in skin tissue engineering, wound healing, wound repair, or cutaneous regeneration, with relevant information on polymer composition, network engineering, bioink performance, biofabrication, bioactivity, or regenerative outcomes. Studies outside this scope or lacking sufficient relevant information were excluded. After duplicate removal, records were screened by title and abstract, followed by full-text assessment of potentially eligible publications. The selection process is summarized in Figure 1.

### 2.3. Data Extraction and Evidence Synthesis

Relevant information on polymer design, network architecture, bioink properties, biological functions, biofabrication strategies, and skin-regenerative applications was extracted. The findings were qualitatively synthesized within a structure–property–function framework linking polymer molecular design and network engineering to bioink performance and regenerative outcomes. This review was reported in accordance with PRISMA 2020 but was not prospectively registered.

### 2.4. Use of Generative AI Tools

During the preparation of this manuscript, ChatGPT (OpenAI, San Francisco, CA, USA, GPT-5.6 Sol) was used to assist with the organization and linguistic refinement of selected textual content. The conceptual frameworks, scientific content, and overall composition of the schematic diagrams below were conceived and designed by the authors. BioRender AI tools were used only to assist in creating selected graphical elements that were not available in the existing BioRender asset library. All AI-assisted elements were subsequently reviewed, modified, and integrated by the authors to ensure scientific accuracy and consistency with the intended concepts. AI tools were not used for literature selection, bibliometric or statistical analyses, data generation, or final scientific interpretation. The authors take full responsibility for the final content of the manuscript.

## 3. Bibliometric Landscape

### 3.1. Publication Trends

**Figure 2:**
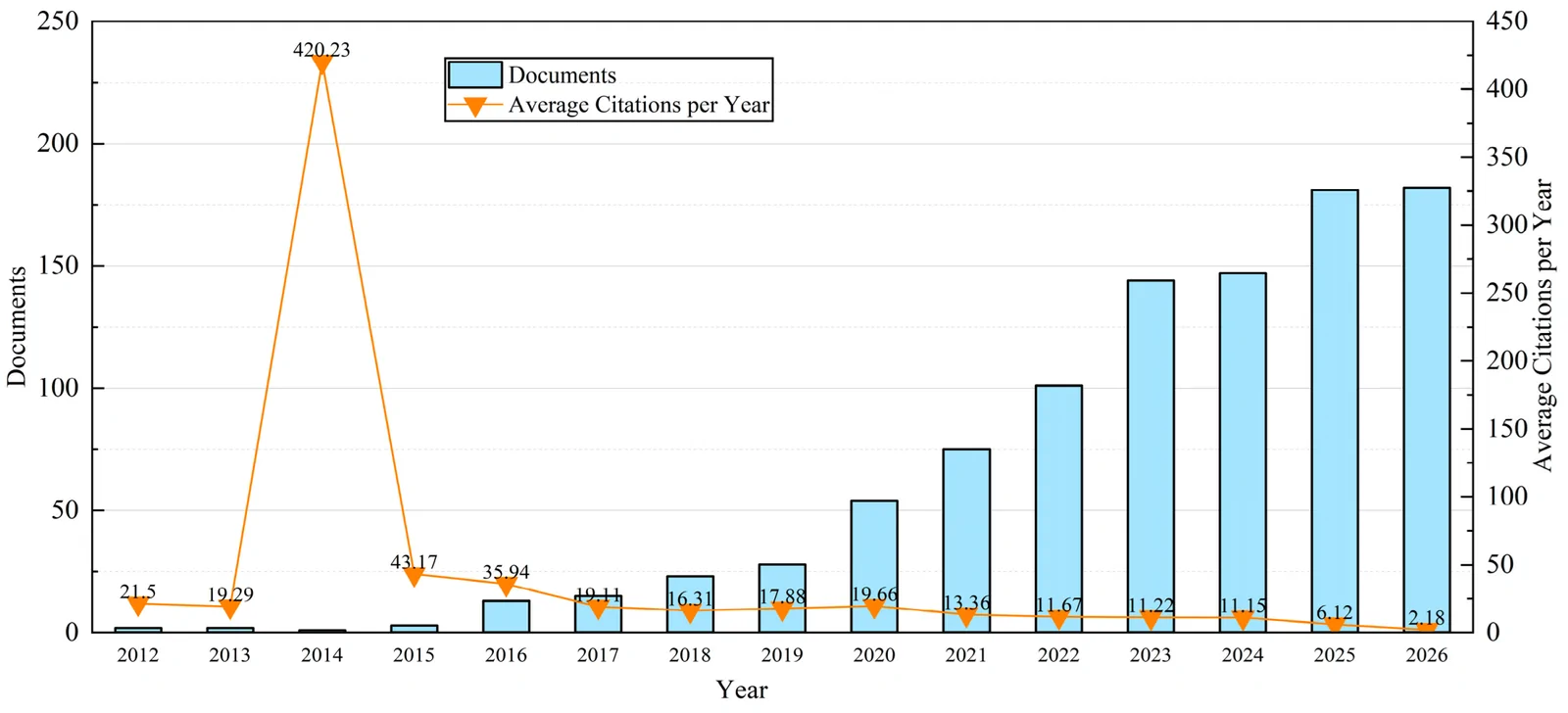
Annual publication output and average citation performance of studies on hydrogel bioinks for skin tissue engineering and wound regeneration from 2012 to 2026.

As shown in Figure 2, research on hydrogel bioinks for skin tissue engineering and wound regeneration has expanded substantially over the past decade. The number of publications on this topic remained small during 2012–2015, increased steadily after 2016, and has grown markedly since 2020. Annual output exceeded 100 publications in 2022, and by 8 September 2026, the number of studies published was already close to the total published in 2025, indicating continued growth of the field. Citation trends showed a different pattern, with the unusually high average in 2014 driven primarily by the highly cited review “3D Bioprinting of Tissues and Organs” [15]. Earlier studies also tend to accumulate more citations because of their longer citation windows. Overall, these trends reflect the transition of hydrogel bioinks from an emerging research topic to a rapidly expanding interdisciplinary field spanning polymer science, biofabrication, and regenerative medicine.

### 3.2. Global Collaboration Network

**Figure 3:**
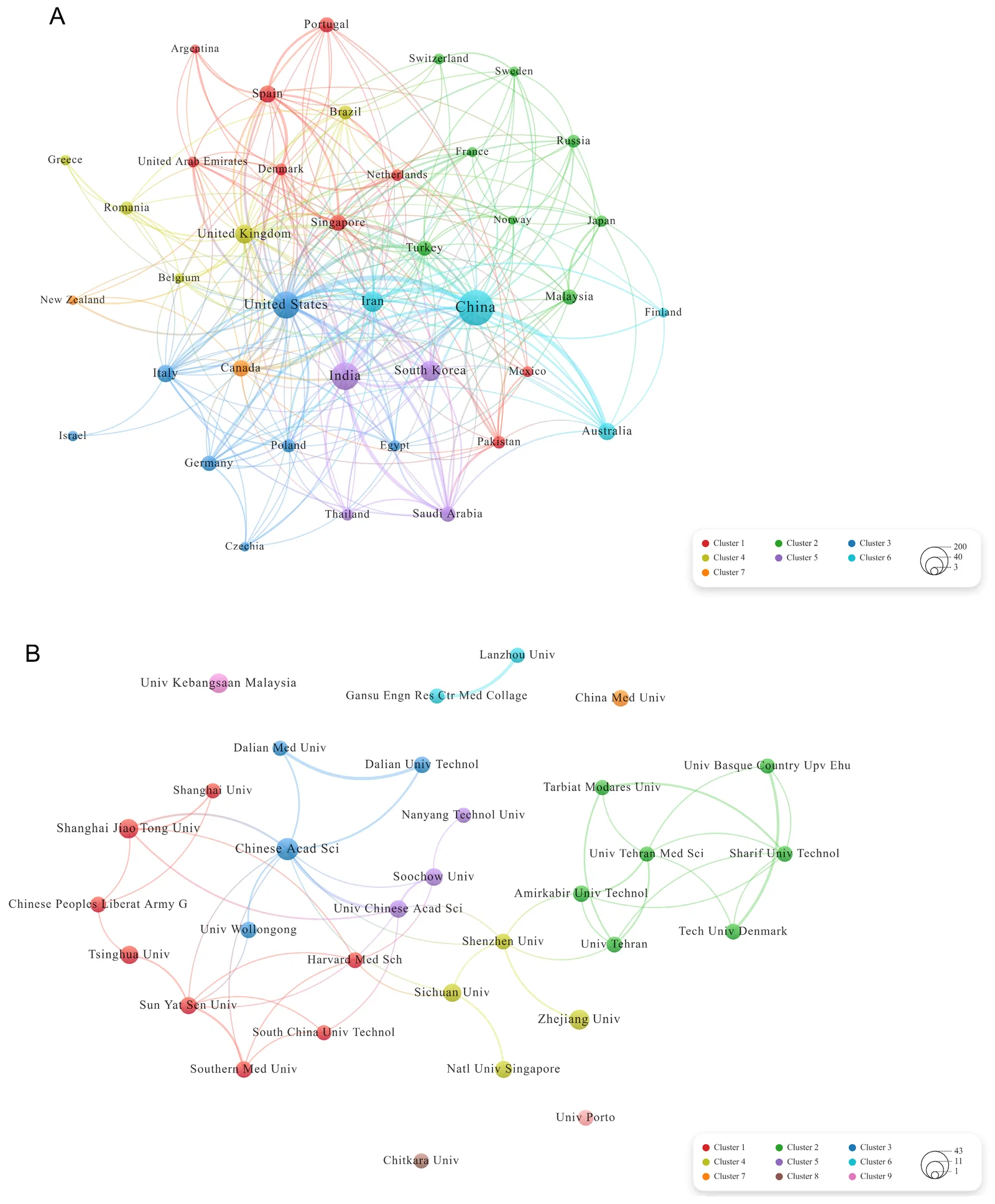
Global collaboration landscape in hydrogel bioink research for skin tissue engineering and wound regeneration. (**A**) Country-level co-authorship network with overlay visualization of average publication year; (**B**) institutional collaboration network. Node size represents relative contribution, while connecting lines indicate collaborative relationships.

The collaboration network demonstrates broad international participation in research on hydrogel bioinks for skin regeneration (Figure 3A,B). China and the United States are the most prominent contributors, with additional participation from Iran, India, the United Kingdom, and other countries. Dense country-level connections indicate an increasingly international research landscape, and the overlay visualization suggests a continuing trend of broad participation in recent years.

At the institutional level, the network exhibits a more clustered structure (Figure 3B). The Chinese Academy of Sciences occupies a central position, alongside several major universities and research institutions within distinct regional subnetworks. Compared with the country-level network, these more localized institutional clusters suggest that stronger cross-institutional collaboration could further promote integration across polymer science, biofabrication, and regenerative medicine.

### 3.3. Journals and Intellectual Knowledge Base

The journal distribution (Table 1) highlights the strongly interdisciplinary nature of research on hydrogel bioinks for skin regeneration. *Nature Biotechnology* showed the highest total citation count (TC = 5463), which was largely attributable to a single highly cited article, whereas the *International Journal of Biological Macromolecules* had the highest publication output (61 articles) and H-index (27). Other influential journals span the topics of biomaterials, polymer science, biotechnology, and biofabrication, highlighting the convergence of material design and regenerative medicine within this field.

The citation structure is similarly shaped by both foundational and recent contributions (Table 2). Murphy and Atala’s 2014 article remains the dominant citation landmark [15], though several more recent studies have achieved high citation rates despite shorter citation windows. Overall, the intellectual base has expanded from a small number of foundational studies toward a broader and more diversified body of literature increasingly focused on polymer engineering and advanced bioink design.

### 3.4. Keyword Evolution and Thematic Transition

**Figure 4:**
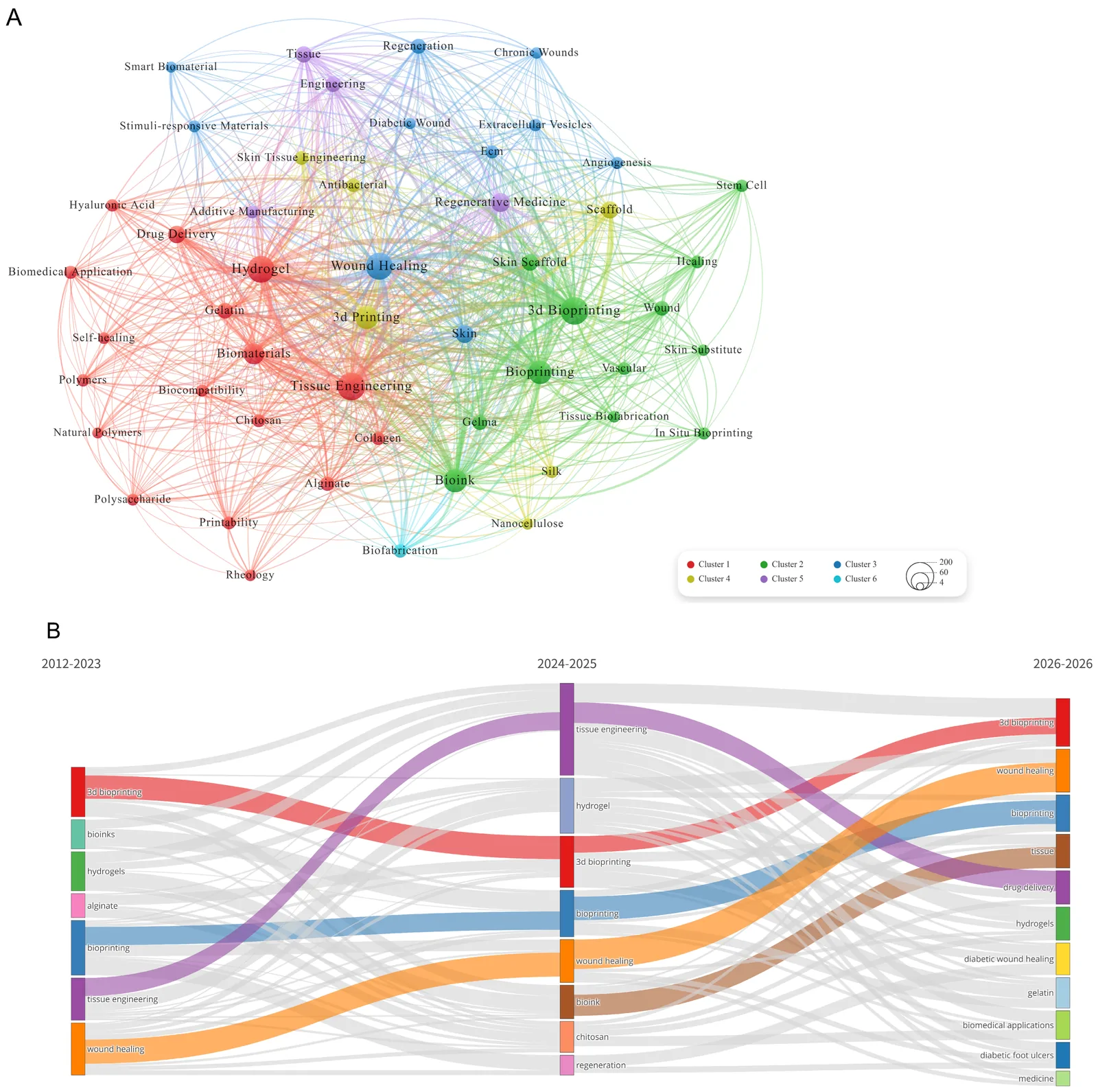
Keyword co-occurrence and thematic evolution in hydrogel bioink research for skin tissue engineering and wound regeneration. (**A**) Keyword co-occurrence network showing major thematic clusters; (**B**) thematic evolution across 2012–2023, 2024–2025, and 2026. Node size represents keyword frequency, and links indicate co-occurrence relationships.

Keyword co-occurrence analysis revealed a highly interconnected research landscape centered on hydrogels, tissue engineering, 3D bioprinting, bioprinting, bioinks, and wound healing (Figure 4A). Material- and processing-related terms remain closely associated with regenerative applications, reflecting the continued importance of polymer design and printability. At the same time, increasing attention to vascularization, chronic wounds, and regenerative medicine indicates a shift from scaffold fabrication toward functional tissue repair. More recent terms related to self-healing, stimuli responsiveness, antibacterial activity, and controlled delivery further suggest the emergence of multifunctional and adaptive bioink systems.

The thematic evolution map provides additional evidence for this transition (Figure 4B). In 2012–2023, research was dominated by material formulation, bioprinting, and wound-healing themes, whereas research from the 2024–2025 period shows greater emphasis on regenerative function. In 2026, topics such as drug delivery and diabetic wound healing became more prominent, although these subjects should be interpreted as emerging directions because this study’s dataset only includes articles published up to and including 8 September 2026.

Overall, these patterns suggest a progressive shift in polymer bioink design from structural matrices focused primarily on fabrication to bioactive matrices supporting regeneration and increasingly toward adaptive systems capable of interacting with the wound microenvironment. The emergence of self-healing, stimuli-responsive, smart biomaterial, and controlled-delivery concepts now indicates a further transition toward adaptive regenerative polymer systems that are designed not only to maintain structure but also to dynamically interact with the wound microenvironment. Thus, the evolution of polymeric bioinks can be broadly conceptualized as the following progression: structural matrices → bioactive matrices → adaptive regenerative systems. This framework provides a conceptual basis for the molecular design, network engineering, and functionalization strategies discussed in the following sections.

## 4. Polymer Molecular Design and Network Architecture

**Figure 5:**
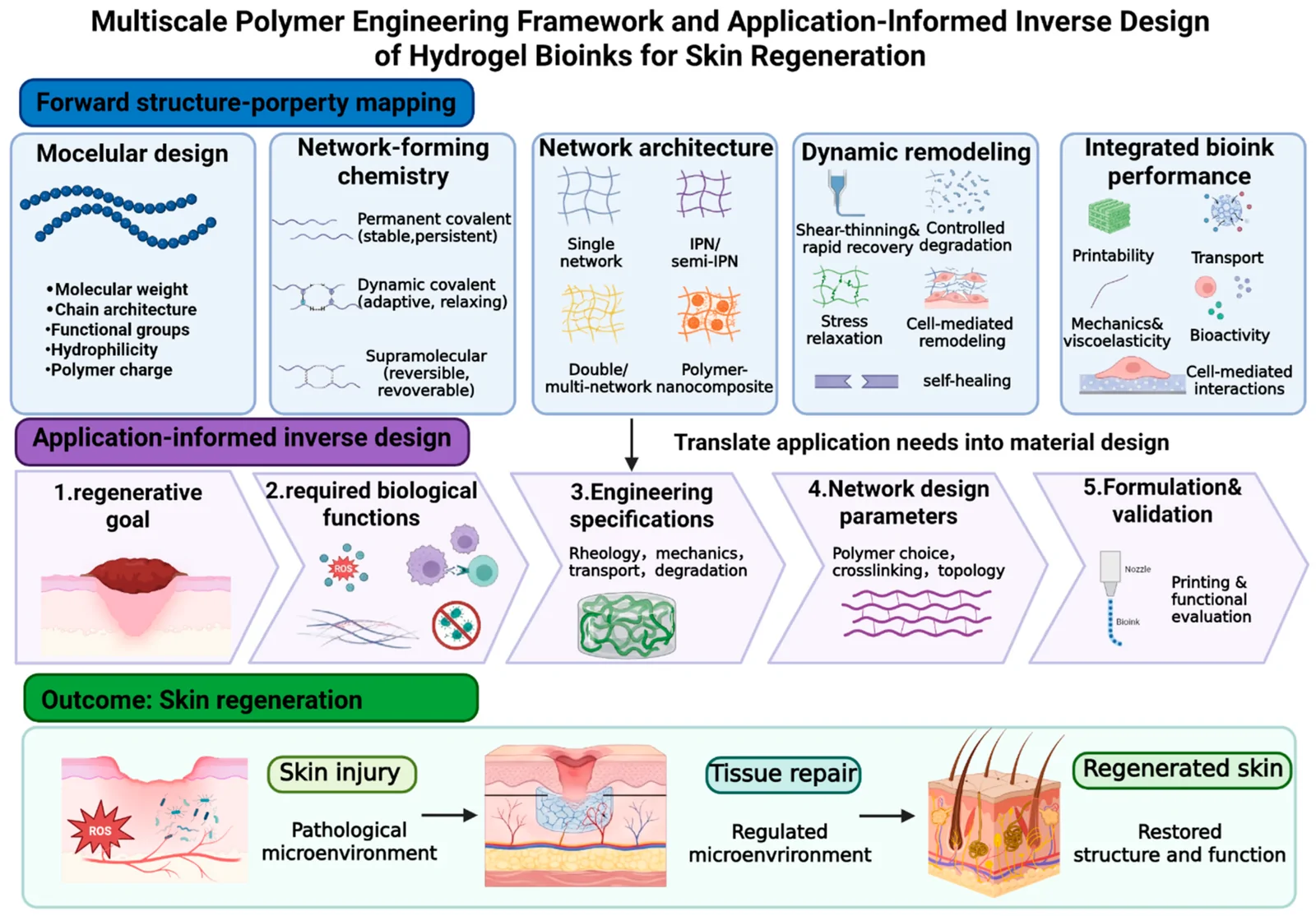
Multiscale polymer engineering framework and application-informed inverse design of hydrogel bioinks for skin regeneration. The upper panel illustrates forward structure–property mapping, linking polymer molecular characteristics, network-forming chemistry, network architecture, and dynamic remodeling to integrated bioink performance, including printability, transport, mechanical and viscoelastic properties, bioactivity, and cell–material interactions. The middle panel presents an application-informed inverse-design pathway, translating regenerative goals and required biological functions into engineering specifications, network-design parameters, and formulation validation. The lower panel illustrates the progression from skin injury and a pathological wound microenvironment to regulated tissue repair and regenerated skin with restored structure and function. IPN, interpenetrating polymer network; ROS, reactive oxygen species. Created in BioRender. Zhu, X. (2026) https://BioRender.com/nr15y7l.

Hydrogel bioink performance is the result of hierarchical interactions between polymer molecular features and network structure. Molecular features determine chain interactions and junction formation, while crosslinking chemistry and network architecture shape the resulting mechanical, transport, and remodeling behavior. These properties ultimately influence cellular organization and regenerative outcomes. Figure 5 summarizes this structure–property–function relationship and highlights the inverse-design perspective adopted in this review, in which regenerative requirements guide the selection of molecular and network parameters.

### 4.1. Molecular Determinants Governing Polymer Bioink Behavior

At the molecular level, a polymer’s molecular weight, chain architecture, concentration, functionalization, hydrophilicity, and charge define the starting conditions for network formation [25]. These variables influence chain entanglement, precursor rheology, and the availability of network-forming sites. Their effects are often coupled, making it important to relate molecular changes to measurable network properties and biological responses rather than considering each parameter in isolation.

Molecular weight and chain architecture primarily affect precursor behavior by changing chain overlap, entanglement, and mobility. These effects are strongly concentration-dependent, which makes it difficult to isolate molecular-weight effects across different polymer systems [26]. For example, increasing the molecular weight of pentenoate-functionalized hyaluronic acid from 1 to 1.5 MDa reduced the polymer concentration required for optimal shape fidelity from 8 to 4 wt%, while both formulations achieved the highest shape-fidelity score [27]. Similarly, at a fixed concentration of 4% (*w*/*w*), higher-molecular-weight alginates exhibited markedly higher zero-shear viscosities and produced more continuous filaments than lower-molecular-weight formulations [28]. These findings suggest that molecular weight primarily shifts the rheological processing window through changes in chain entanglement and precursor viscosity rather than simply improving printability monotonically.

Polymer concentration provides another clear example of how molecular parameters propagate across multiple material properties. In a GelMA–Carbopol system with the Carbopol concentration fixed at 0.5%, increasing GelMA from 3% to 7% increased the printability parameter from 0.30 ± 0.10 to 0.72 ± 0.03. At the lower GelMA concentration, extruded filaments underwent substantial broadening and deformation, whereas higher concentrations produced better-defined and less deformable filaments [29]. In a GelMA system with consistent methacrylation, increasing the concentration from 5% to 20% (*w*/*v*) raised the compressive modulus from 3 ± 1 to 108 ± 8 kPa and reduced the swelling ratio from 1476 ± 28% to 470 ± 9%11. The 5% hydrogel degraded completely within 3 days, whereas approximately 20% of the 20% GelMA matrix remained after 56 days. HaCaT viability remained above 90%, while cell attachment and proliferation increased with the GelMA concentration [20]. These findings show that concentration simultaneously reshapes mechanics, hydration, degradation, and cellular behavior rather than controlling a single property.

Reactive-group density provides a direct means of controlling network formation. In GelMA, Chen et al. [30] increased the degree of methacrylation from 49.8% to 63.8% and 73.2%. The corresponding compressive modulus increased from 2.0 ± 0.18 to 3.2 ± 0.18 and 4.5 ± 0.33 kPa, while the average pore size decreased from 49.7 ± 11.8 to 30.13 ± 6.12 and 23.6 ± 5.85 μm. Collagenase-mediated degradation was prolonged from approximately 6 h to 15 h. Greater methacrylation was also associated with poorer ECFC survival, reduced MSC proliferation, and weaker vascular-network formation. After implantation, microvessel density decreased from 58.34 ± 32.70 vessels mm^−2^ in the low-methacrylation group to 7.20 ± 9.15 vessels mm^−2^ in the highly methacrylated group. Thus, greater functionalization increased network stiffness and persistence at the expense of cellular remodeling and vascularization.

Hydrophilicity and charge further modulate hydration and biomolecule–matrix interactions. Their biological effects, however, depend strongly on the surrounding polymer chemistry and ionic environment, making them context-dependent modifiers rather than independent predictors of bioink performance [31].

Molecular design therefore defines the range of network states available to a bioink but cannot determine its final behavior alone. The next level of control is network-forming chemistry, which translates these molecular features into differences in mechanics, transport, and network dynamics.

### 4.2. Network-Forming Chemistry

Crosslinking chemistry converts precursor polymers into three-dimensional networks and determines gelation kinetics, bond reversibility, and network rearrangement. During printing, network formation must balance flow during deposition with rapid stabilization after printing. Different crosslinking mechanisms, including permanent covalent, dynamic covalent, and supramolecular crosslinks, provide distinct trade-offs between structural stability and network adaptability.

#### 4.2.1. Permanent Covalent Crosslinking

Permanent covalent crosslinking provides structural stability through near-irreversible bond formation. Methacrylate, acrylate, and norbornene functionalities are widely used to establish covalent hydrogel networks [32], while alternative coupling reactions can enable crosslinking under mild aqueous conditions. The main advantage of these networks is their ability to maintain structural integrity after fabrication. However, increasing the crosslink density often alters several network properties simultaneously, making it difficult to attribute biological responses to stiffness alone.

The independent role of mesh architecture was examined using polyacrylamide substrates with comparable apparent stiffnesses [33]. By adjusting the polymer concentration and hydrogel-layer thickness, substrates of approximately 13 kPa were generated with different mesh structures. Human bone marrow-derived mesenchymal stem cells (hBMSCs) exhibited greater spreading, faster migration, larger focal adhesions, and increased nuclear localization of yes-associated protein (YAP) on substrates with smaller meshes, whereas initial cell attachment was not significantly affected. These results demonstrate that mesh architecture can influence cell–material interactions independently of apparent stiffness.

A different response was observed when matrix stiffness and mesh size varied together in a GelMA/PEGDA system [34]. With GelMA maintained at 5% (*w*/*v*), increasing PEGDA from 2.5% to 10% (*w*/*v*) raised the compressive modulus from 1.3 ± 0.5 to 23.0 ± 1.0 kPa while reducing the calculated mesh size from 15 ± 0.6 to 11 ± 0.2 nm. Fibroblast spreading increased accordingly. In contrast, endothelial sprouting showed a non-monotonic response, increasing at intermediate degrees of stiffness before declining at higher ones. Because stiffness and mesh size varied concurrently, their individual contributions cannot be resolved from this system alone.

Together, these studies show that covalent network design cannot be reduced to maximizing stiffness. Mesh architecture and matrix mechanics may regulate distinct biological responses, and their effects can become coupled as crosslink density changes. For skin-regenerative bioinks, permanent covalent crosslinking should therefore be tuned to the intended regenerative function rather than optimized solely for structural persistence.

#### 4.2.2. Dynamic Covalent Crosslinking

Dynamic covalent networks rely on reversible bonds that dissociate and reform while preserving overall network connectivity. Representative chemistries include imine or hydrazone bonds and boronate esters [35]. Unlike permanent networks, their behavior depends not only on crosslink density but also on bond-exchange kinetics. Faster exchange promotes network rearrangement and recovery during extrusion, whereas slower exchange favors structural persistence but may limit stress relaxation and cellular remodeling. The importance of exchange kinetics was demonstrated in hydrazone-crosslinked hyaluronan–elastin-like protein (HELP) bioinks [36]. A 1 wt% HA-ALD/1 wt% ELP-HYD network exhibited a storage modulus of approximately 10^3^ Pa. The addition of a small-molecule competitor reduced the modulus by up to two orders of magnitude, while formulations containing 20 mM competitor still gelled within 30 min. With a 10 mM competitor, the initial modulus decreased approximately fourfold but recovered to the level of competitor-free gels after 24 h in PBS as the competitor diffused from the network. In parallel, a catalyst accelerated hydrazone exchange and enhanced shear-thinning behavior. These results demonstrate that transient network connectivity and exchange kinetics can be tuned to facilitate extrusion while preserving post-printing mechanical recovery.

Dynamic covalent network design should therefore not aim to maximize reversibility; instead, it should establish a kinetic window that enables sufficient rearrangement during printing while retaining the structural persistence and adaptability required for subsequent regeneration.

#### 4.2.3. Supramolecular Crosslinking

Physical and supramolecular networks are formed through reversible non-covalent interactions, including ionic association and molecular recognition [37]. These interactions can support shear thinning during extrusion and structural recovery after deposition without requiring extensive permanent bond formation. Their performance depends on the strength and reversibility of the interactions, as well as the surrounding environment, making printability and post-printing stability highly context-dependent. A representative hybrid strategy combines ionic and covalent crosslinking in GelMA–alginate bioinks [38]. Within a GelMA–alginate concentration range of 11–15% (*w*/*v*), suitable printing performance was achieved, while selected formulations showed less than 20% degradation and negligible swelling over 14 days, with post-printing cell viability remaining above 75%. The combined network also enabled stripe-patterned constructs with direction-dependent mechanical properties. These findings illustrate that supramolecular interactions cannot be evaluated independently of the surrounding covalent network and formulation context. The suitability of a supramolecular crosslinking system should therefore be evaluated according to its combined printing, structural, and biological performance rather than a single rheological or mechanical metric. Crosslinking chemistry defines the behavior of individual junctions, whereas their spatial organization and integration with other polymer phases determine the network architectures discussed in Section 4.3.

### 4.3. Network Topology and Hierarchical Architectures

Crosslinking chemistry defines the nature of individual network junctions, whereas network topology determines how these junctions and polymer phases are organized. This organization influences mechanics, transport, rheology, and cellular remodeling. In conventional single-network hydrogels, fabrication and biological requirements are often coupled, so improving one property may compromise another. Hierarchical architectures can reduce this constraint by distributing different functions across distinct structural components. Interpenetrating, multi-network, and composite systems therefore provide a broader design space for balancing printability, stability, and regenerative performance.

#### 4.3.1. Interpenetrating and Semi-Interpenetrating Polymer Networks

Interpenetrating polymer networks (IPNs) contain two independently crosslinked networks that are physically interlaced [39]. Semi-interpenetrating polymer networks (semi-IPNs), in contrast, contain one crosslinked network penetrated by non-crosslinked polymer chains [40]. These architectures allow different polymer phases to perform complementary roles. This functional complementarity was demonstrated in a Gel-HDA/GelMA IPN designed for extrusion bioprinting [41]. The IPN exhibited a sol–gel transition temperature of 30 °C compared with 27 °C for GelMA. Varying the Gel-HDA/GelMA ratio adjusted the compressive modulus from 20.6 ± 2.48 to 116.7 ± 14.80 kPa and altered room-temperature printability, while encapsulated MC3T3-E1 (mouse calvarial pre-osteoblast) cells maintained high viability and proliferated over the course of 10 days. These findings show that IPN composition can expand the accessible processing and mechanical window without relying on excessive crosslinking of a single network. However, functional complementarity requires compatibility between the component networks, as mismatched swelling or degradation may introduce structural heterogeneity. IPN design should therefore prioritize coordinated network behavior rather than simply increasing architectural complexity.

#### 4.3.2. Double-Network and Multi-Network Architectures

Double-network (DN) hydrogels combine networks with complementary mechanical roles, typically coupling energy dissipation with structural support. This architecture can improve toughness and post-printing stability without requiring uniformly high crosslink density [42]. The benefit of secondary network formation was demonstrated in a printable dynamic covalent hydrogel reinforced with a photocrosslinkable HA network [43]. Photostiffening increased the modulus by approximately 300% while reducing scaffold erosion by approximately 70%, illustrating how sequential network formation can strengthen printed constructs without replacing their initial dynamic character. Using a related strategy, elastin was incorporated into a GelMA/HA-based DN system, producing a hydrogel with approximately 170% tensile strain, a toughness of 45.39 kJ m^−3^, and a tolerance of about 80% compressive deformation without rupture [38]. These results illustrate how complementary networks can combine deformability, energy dissipation, and structural reinforcement.

However, the addition of networks also increase formulation complexity and may constrain transport or cellular remodeling. DN and multi-network design should therefore prioritize functional complementarity rather than the number of networks, introducing additional ones only when they provide a clear fabrication or regenerative benefit.

#### 4.3.3. Polymer–Nanocomposite and Hierarchical Networks

Polymer–nanocomposite networks introduce nanoscale components into polymer matrices to generate additional structural and functional interactions [44]. These interactions can reinforce network structure and improve rheological recovery without requiring uniformly higher covalent crosslink density. Their effectiveness, however, depends strongly on particle dispersion and polymer–particle interactions. Chimene et al. demonstrated this principle using a nanoengineered ionic–covalent entanglement (NICE) bioink composed of GelMA, κ-carrageenan, and nanosilicates [45]. The NICE network reached a compressive modulus of 71.1 ± 4.9 kPa, compared with 16.5 ± 1.5 kPa for GelMA alone. Its storage modulus increased from 3.1 ± 0.3 to 14.2 ± 1.4 kPa. The printed constructs also maintained >85% cell viability after extrusion. The above results prove that nanoscale reinforcement can work synergistically with polymer networks rather than simply acting as a passive filler. Nanocomposite bioinks should therefore be optimized according to interfacial interactions and particle distribution, since excessive or heterogeneous nanoparticle loading may reduce reproducibility and biological compatibility.

### 4.4. Network Dynamics and Remodeling

The quantitative relationships summarized in Table 3 primarily describe how molecular composition, crosslinking chemistry, and network architecture define the accessible material states of hydrogel bioinks. However, these states are not static. Network junctions and polymer chains can rearrange, relax, dissociate, reform, or degrade over time, introducing an additional temporal dimension to bioink design. Network dynamics describe the time-dependent rearrangement and remodeling of polymer networks. This temporal behavior is particularly important for bioinks because their functional requirements change during regeneration: sufficient stability is needed during fabrication, whereas later stages require increasing matrix permissiveness for cellular remodeling and tissue formation.

Stress relaxation is a key dynamic parameter and can regulate cell behavior independently of initial stiffness [37]. In a seminal alginate hydrogel system, Chaudhuri et al. [46] reduced alginate’s molecular weight from 280 to 35 kDa and introduced 5 kDa PEG spacers, shortening the stress relaxation half-time from approximately 1 h to 1 min while maintaining the initial modulus, degradation, and ligand density. At an initial modulus of approximately 9 kPa, faster relaxation significantly increased fibroblast spreading and proliferation (*p* < 0.0001), while rapidly relaxing matrices at 17 kPa supported the formation of a mineralized, collagen I-rich matrix. Stress relaxation’s relevance to vascular remodeling was further demonstrated in ELP–PEG hydrogels, where fast-relaxing matrices promoted endothelial spreading and vascularization compared with stiffness-matched slow-relaxing networks [48].

Network dynamics also influence fabrication because reversible junctions can recover after extrusion, while subsequent degradation or cell-mediated remodeling progressively increases matrix accessibility. However, excessive relaxation can compromise structural stability. Bioink design should therefore establish a temporal window that supports early shape retention and later biological remodeling rather than maximizing network dynamics at all stages.

### 4.5. Polymer Platforms for Hydrogel Bioinks

The molecular and network design principles discussed above can be implemented using natural, synthetic, or hybrid polymer platforms. Natural polymers generally offer biologically recognizable motifs, enzymatic degradability, and ECM-like environments, while synthetic polymers offer more precise control over molecular structure, crosslinking, and reproducibility. Neither class independently satisfies all requirements of advanced biofabrication (Table 4). Consequently, polymer selection should be based on the specific biological and fabrication functions required rather than on material origin alone.

#### 4.5.1. Natural Polymer Platforms

Natural polymers remain central to hydrogel bioink development because many possess structural or biochemical features related to native ECM components. Protein-derived polymers such as gelatin, collagen, and fibrin contain molecular motifs that support cell adhesion, migration, and matrix remodeling, whereas polysaccharides such as hyaluronic acid (HA), alginate, and chitosan provide distinct hydration, charge, and functional-group characteristics. However, their biological advantages are often accompanied by limited mechanical stability, rapid degradation, or insufficient printability, making additional network engineering necessary.

One strategy is to introduce complementary bio-derived components to improve matrix performance. In a gelatin-based system containing Aloe vera and other natural additives, optimized formulations reduced degradation from 5.36% to 1.99%, maintained cell viability up to 92.46%, and achieved a wound-closure rate of 66.15% [64]. These results illustrate how bio-derived components can improve material performance while retaining biological activity.

A decellularized extracellular matrix (dECM) provides a more complex source of tissue-derived biochemical cues. When incorporated into a fibrinogen-based bioink, a dECM increased the storage modulus from 116.5 ± 4.4 to 780.3 ± 22.6 Pa and improved construct stability during printing [65]. After 15 days, cell viability reached 73.8 ± 3.7% in dECM-containing constructs compared with 55.3 ± 2.8% in the fibrinogen control. This example shows that tissue-derived matrix components can enhance both structural performance and long-term cell support when integrated into a complementary polymer network.

Natural polymer platforms are therefore highly biologically relevant, but they generally require additional control over network formation and stability for reproducible biofabrication. Their design should preserve useful biological cues while introducing only the structural reinforcement needed for the intended application.

#### 4.5.2. Synthetic Polymer Platforms

Synthetic polymers provide a complementary design space in which molecular weight, chain architecture, functional-group density, and degradation mechanisms can be precisely controlled [25]. Representative platforms include PEG-based polymers, PVA, and thermoresponsive block copolymers; their major limitation is a lack of intrinsic biological recognition, which often requires additional biofunctionalization [66].

The programmability of synthetic polymers is illustrated by a PEG-based thermoresponsive triblock copolymer developed as a cell-laden bioink [67]. At 35% (*w*/*w*), the material exhibited a gel-transition temperature of approximately 8 °C and supported printing at 450 mm min^−1^ at 37 °C. It also showed lower cytotoxicity than Pluronic F127 under comparable conditions and enabled direct printing of encapsulated human mesenchymal stem cells. This example demonstrates how polymer composition and architecture can be engineered to control thermal response and printability.

PEG further exemplifies the modularity of synthetic platforms because its network properties can be tuned through controlled modification with reactive or degradable groups^120^. Its bioinert nature, however, generally necessitates the incorporation of adhesive or matrix-responsive cues for regenerative applications [68]. PVA and Pluronic F127 offer complementary advantages in network formation and rheological control, although their limited intrinsic cell interactions similarly restrict their use as standalone regenerative matrices [69].

The principal value of synthetic polymers therefore lies in molecular programmability rather than inherent biological activity. Their future development should combine this physicochemical precision with sufficient biological functionality and remodeling capacity while avoiding unnecessarily complex formulations.

#### 4.5.3. Hybrid Polymer Platforms

Hybrid polymer platforms combine natural and synthetic components to integrate biological functionality with greater control over processing and network properties [63]. Hybridization therefore enables different polymer components to perform distinct functions within a shared material system.

For example, Masri et al. developed a gelatin–PVA hybrid bioink for skin tissue engineering. Increasing PVA from 0 to 5% (*w*/*v*) in a 6% gelatin formulation increased its viscosity to approximately 3.0 ± 0.06 Pa·s and improved extrusion printability. After genipin crosslinking, the optimized formulation maintained >90% viability of human dermal fibroblasts while providing a stable porous structure [70]. These findings show that synthetic reinforcement can improve processing without eliminating the biological compatibility of the natural polymer phase.

Importantly, hybridization should not be treated as a simple blending of materials. Its effectiveness depends on controlled interactions and compatibility between the constituent networks because mismatched network formation or remodeling may generate heterogeneous structures. Each component should therefore have a defined functional role within the hybrid system.

**Figure 6:**
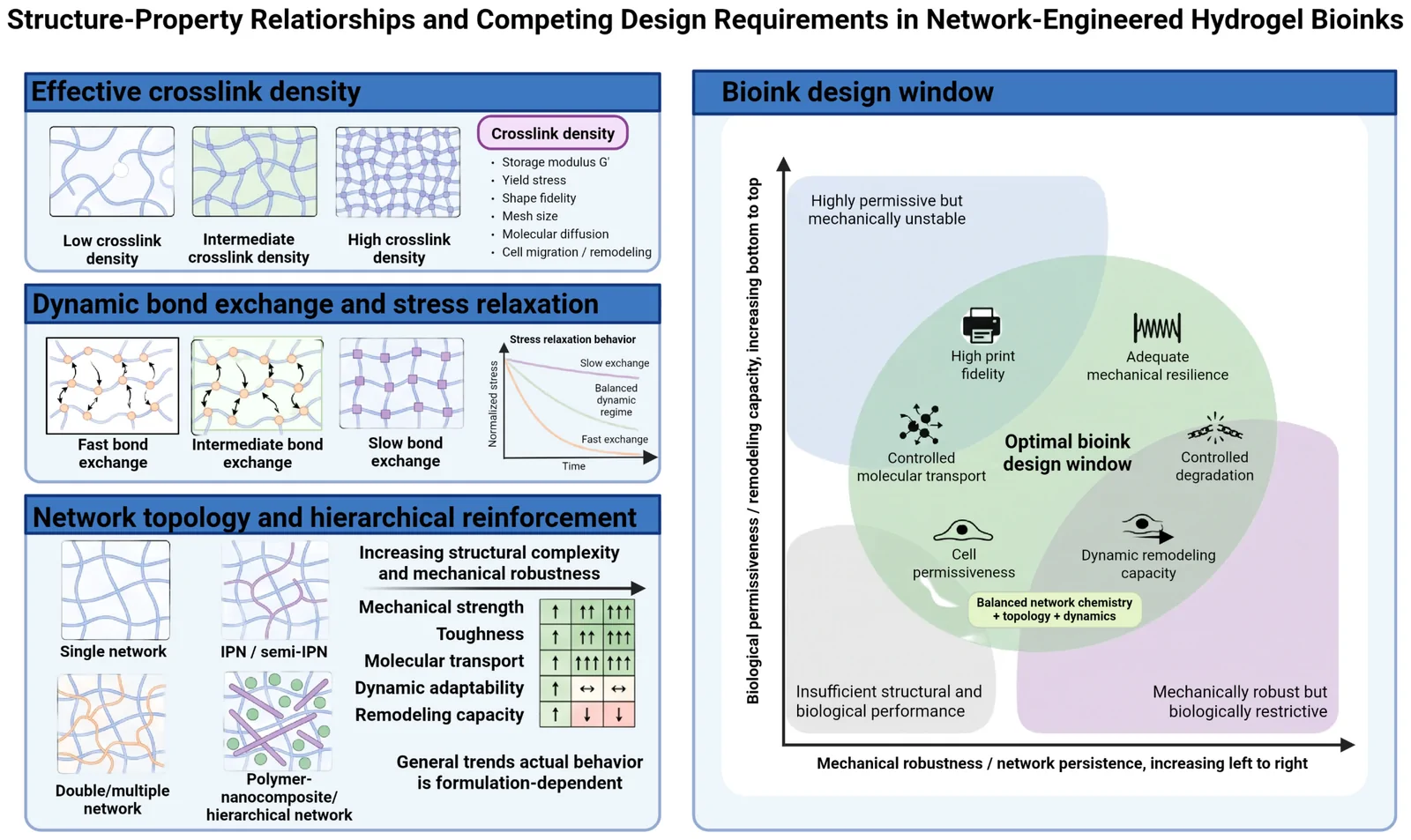
Structure–property relationships and competing design requirements in network-engineered hydrogel bioinks. Crosslink density, bond-exchange kinetics, and network topology jointly regulate mechanical stability, molecular transport, stress relaxation, and remodeling capacity. Optimal bioink performance requires balancing mechanical robustness with biological permissiveness and dynamic remodeling rather than maximizing any single property. IPN, interpenetrating polymer network. Created in BioRender. Zhu, X. (2026) https://BioRender.com/pw3lv9b.

Despite the rapid expansion of polymer chemistries and network-engineering strategies, a broader limitation of the existing literature is that improved bioink performance is often attributed to “network optimization”, even when several molecular and formulation variables change simultaneously. This makes it difficult to identify the dominant mechanism responsible for improved printing or regenerative performance. In our view, future studies should increasingly decouple these variables and establish quantitative structure–property–function relationships rather than relying on formulation-by-formulation comparisons. Such mechanistic studies are essential for moving polymer bioink design from empirical optimization toward predictive engineering. As summarized in Figure 6, these relationships can be conceptualized as a design window in which crosslink density, bond-exchange kinetics, and network topology are balanced against mechanical robustness, molecular transport, cellular permissiveness, and remodeling capacity.

## 5. Multidimensional Property Engineering of Polymer Bioinks

The performance of hydrogel bioinks is governed by multiple interdependent properties, including rheology, mechanics, transport, degradation, cell–material interactions, and bioactivity. Because optimizing one parameter may compromise others, experimentally supported property relationships and trade-offs are examined using representative quantitative evidence from the literature.

### 5.1. Rheology and Printability

Rheological behavior determines whether a bioink can be extruded reproducibly while retaining sufficient structural stability after deposition. Accordingly, printability should be evaluated by linking shear-dependent rheology with quantitative outcomes such as filament continuity, dimensional fidelity, and post-print stability [71].

Rahimnejad et al. demonstrated this relationship using thermosensitive chitosan inks containing 2% (A2) or 3% (A3) chitosan. After exposure to 100 s^−1^ shear, the immediate post-shear viscosity of A3 was 663 ± 95 Pa·s, approximately tenfold higher than that of A2 (66 ± 15 Pa·s). A2 produced broad filaments of 1.2–1.5 mm, whereas A3 produced thinner filaments (0.57 ± 0.04 mm at 11 mm/s) that became continuous when the feed speed was reduced to 10 mm/s (0.75 ± 0.07 mm). Shear history also affected construct mechanics, with the secant Young’s modulus of A3 decreasing from 24.4 ± 0.7 to 4.3 ± 0.1 kPa as the shear rate increased from 230 to 507 s^−1^. Thus, polymer concentration, shear history, and printing speed jointly determine printing fidelity and post-print stability [71].

A skin-specific processing range was reported by Ullah et al. for a PEO–chitosan–PMMA bioink. At 37 °C, reproducible extrusion occurred at 25–50 kPa within a viscosity range of 500–550 Pa·s, while cell survival reached 92% compared with 67% for pure chitosan [72]. This data further illustrates that printability depends on matching material rheology with extrusion conditions rather than simply maximizing viscosity.

Network reinforcement can improve shape retention but may compromise other properties. Hu et al. tuned the Young’s modulus of a dual physically crosslinked supramolecular bioink from approximately 4 to 130 kPa by increasing the β-glycerophosphate concentration from 0.3 to 1.3 M. Dual-crosslinked constructs maintained their geometry for at least 21 days, whereas singly crosslinked constructs deformed shortly after printing [73]. However, greater reinforcement was accompanied by reduced pore size and porosity. Similarly, Mendoza-Cerezo et al. found that reducing GelMA content from 100% to 75% and then 50% increased the collapse coefficient at a 7.5 mm unsupported span from 1.10% to 8.20% and then 15.10%, respectively, despite comparable 24 h cell viabilities of 76.1–80.8%. The 75% GelMA formulation therefore provided a more favorable balance between cytocompatibility and structural fidelity [49].

Taken together, these studies show that printability is the product of the coupled effects of polymer concentration, shear response, crosslinking, and processing conditions. Higher viscosity or stiffness can improve structural fidelity but may compromise filament continuity, porosity, or biological accessibility; moreover, differences in nozzle geometry, rheological protocols, and crosslinking conditions limit direct comparison across studies.

### 5.2. Mechanical and Viscoelastic Performance

The elastic modulus cannot fully describe the mechanical behavior of hydrogel bioinks alone. Toughness, extensibility, viscoelasticity, and remodeling capacity collectively determine whether a construct can maintain its structural integrity while remaining permissive to cellular migration and matrix reorganization. Thus, increasing network strength may improve mechanical stability but simultaneously reduce biological adaptability. Palierse et al. showed that increasing the concentration of porcine skin-derived dECM from 1 to 5 mg mL^−1^ increased the storage modulus from approximately 10 to 1000 Pa while markedly suppressing fibroblast infiltration in the denser 5 mg mL^−1^ matrix [74]. This inverse relationship illustrates that greater viscoelastic stability does not necessarily translate into a more cell-permissive microenvironment.

A comparable trade-off was documented by Jiao et al. for alginate/gelatin bioinks. Increasing gelatin loading from 8 to 12 wt% elevated the compressive modulus from 25.6 ± 2.7 to 37.9 ± 3.5 kPa and elongation at break from 57.9 ± 4.2% to 92.1 ± 1.3%. At the same time, however, the 14-day degradation ratio decreased from 62.8 ± 2.9% to 26.4 ± 2.4%, while the pore size declined from 128.5 ± 16.6 to 79.4 ± 19.7 μm. The intermediate A4G10 formulation achieved compressive and tensile moduli of 32.2 ± 4.1 and 31.7 ± 3.9 kPa, respectively, alongside ~98% cell viability [75]. Rather than favoring the stiffest formulation, these results support an intermediate mechanical range that preserves both structural support and matrix remodeling capacity.

The mechanical behavior of bioinks should also be considered over time rather than only at the point of fabrication. Hydration, degradation, network relaxation, and tissue ingrowth can progressively alter stiffness and load-bearing capacity. Accordingly, characterization should extend beyond the initial modulus to include the evolution of elasticity, deformation tolerance, and structural recovery under physiologically relevant conditions.

### 5.3. Transport, Swelling, and Degradation

Transport, swelling, and degradation are interdependent consequences of network composition, pore architecture, and matrix stability. Together, they determine whether a bioink can maintain its hydration and molecular accessibility while preserving sufficient structural integrity during tissue formation.

This coupling is evident in GelMA–HAMA and adipose-derived decellularized extracellular matrix (adECM)–GelMA–HAMA bioinks used for full-thickness skin wound repair. In the GelMA–HAMA system, the measured swelling index rose from 3.94 ± 0.32 at 1 h to 8.95 ± 0.72 at 24 h. The adECM–GelMA–HAMA bioink exhibited corresponding swelling values of 4.89 ± 0.34 and 11.18 ± 0.45 at the same time points. After 72 h of collagenase digestion, 53.06 ± 5.16% of the adECM-laden hydrogel remained intact versus 37.53 ± 4.52% for GelMA–HAMA. Adding the adECM also enlarged the average pore diameter from 112.16 ± 8.07 to 131.39 ± 6.88 μm [76]. These results show that the same compositional modification simultaneously increased water uptake, pore size, and resistance to enzymatic degradation, illustrating the difficulty of tuning transport-related properties independently.

A different pattern was observed in gelatin/alginate bioinks containing graded gelatin. Cavallo et al. compared GEL-10-ALG, GEL-15-ALG, and GEL-20-ALG formulations crosslinked with 100–200 mM CaCl_2_ and found less swelling in GEL-15-ALG and GEL-20-ALG than in GEL-10-ALG. Degradation also varied nonlinearly: mass loss began around day 10 for GEL-10-ALG but not until day 17 for GEL-15-ALG and GEL-20-ALG. By day 35, GEL-20-ALG had degraded completely, whereas approximately 20% and 60% of the initial mass of GEL-10-ALG and GEL-15-ALG, respectively, remained. Changing the CaCl_2_ concentration within the tested range had little effect on either swelling or degradation, and the intermediate GEL-15-ALG formulation showed the greatest long-term mass retention [77]. These findings indicate that degradation cannot always be predicted directly from polymer loading or crosslinker concentration.

In contrast, a clear concentration-dependent relationship was reported for single-component GelMA bioinks. After 72 h of collagenase exposure, 16 ± 2.0%, 43 ± 0.1%, and 64 ± 1.6% of the mass of 5%, 10%, and 15% GelMA remained, respectively, while the 5% formulation was completely degraded by day 7. In this system, increasing the GelMA concentration progressively improved resistance to enzymatic degradation [78], in contrast to the non-monotonic behavior observed in the gelatin/alginate formulations.

Collectively, these studies show that swelling, pore accessibility, and degradation are formulation-dependent and do not follow a universal linear relationship with polymer concentration. A compositional change may simultaneously alter hydration, pore architecture, and matrix persistence, but the formulation with the highest polymer loading does not necessarily exhibit the slowest degradation. Because degradation media, crosslinking conditions, and measurement protocols differ substantially among studies, absolute values should not be compared directly across systems. Bioink design should therefore rely on formulation-specific relationships among water uptake, network accessibility, and degradative stability rather than on a single material parameter.

### 5.4. Cell–Material Interactions

Cell–material interactions determine whether the encapsulated cells merely survive or can actively adhere, spread, proliferate, and remodel their surroundings. These responses are jointly regulated by biochemical ligand presentation and the physical dynamics of the polymer networ.

The importance of biochemical adhesion is illustrated by RGD-functionalized alginate bioinks containing adult human dermal fibroblasts. Zhu et al. found that arginine–glycine–aspartate (RGD) modification maintained cell viability and promoted integrin clustering, whereas viability in unmodified 2% alginate progressively declined over 28 days. At day 5, fibroblast proliferation was higher in 5% than in 2% RGD-functionalized alginate (0.19 ± 0.03 vs. 0.14 ± 0.01, *p* = 0.001) and increased to 0.24 ± 0.05 by day 28; in comparison, the corresponding values for unmodified 2% and 5% alginate were 0.18 ± 0.01 and 0.17 ± 0.01, respectively [79]. These findings reveal that ligand functionalization can convert a relatively bioinert network into a more cell-interactive matrix, while viability alone may underestimate differences in cellular functionality.

Network dynamics provide an additional level of regulation independent of ligand density. Using double-crosslinked pectin bioinks containing a fixed RGD concentration of 2 mM, Bebiano et al. showed that increasing Ca^2+^ from 6 to 10 mM shortened the stress relaxation time from approximately 470 to 248 s while the elastic modulus changed only modestly from 910.3 ± 156.2 to 1347.2 ± 366.1 Pa. Fibroblasts in the faster-relaxing matrices spread within approximately 24 h and subsequently formed interconnected networks, whereas spreading remained restricted in slower-relaxing matrices. A separate ligand-density experiment further showed that increasing RGD from 0.5 to 2 mM enhanced fibroblast adhesion and spreading [80]. Together, these observations indicate that adhesive signaling and viscoelastic relaxation regulate cellular behavior through complementary rather than interchangeable mechanisms.

Accordingly, cytocompatibility should not be determined according to short-term viability alone. Evaluation of bioink–cell interactions should also include proliferation, adhesion, morphology, spreading, and matrix remodeling to determine whether the material supports active cellular organization rather than passive survival.

### 5.5. Bioactivity and Spatiotemporal Signal Presentation

Bioactivity extends the function of hydrogel bioinks beyond structural support by enabling polymer networks to actively regulate cellular behavior and the local regenerative microenvironment. This function depends not only on incorporating regenerative factors but also on controlling their retention, release kinetics, and spatial presentation within the polymer network [81]. Effective signal delivery therefore requires avoiding both rapid burst release and excessive factor retention and should be evaluated using quantitative measures such as release duration, cumulative release, and spatial release profiles.

Affinity-based signal retention is illustrated by a heparin-functionalized bioink containing 0.3% hyaluronic acid, 10% GelMA, and 0.5% HepMA. Li et al. reported that covalently incorporated HepMA supported sustained VEGF release for up to 21 days [82]. demonstrating how polymer–growth factor affinity can prolong local signal availability within printed constructs.

Spatial architecture provides a distinct mechanism for controlling release. In 3D-printed heparin hydrogels, Wang et al. used a core–shell configuration to regulate the sequential delivery of VEGF and PDGF. When VEGF was loaded in the shell and PDGF in the core, VEGF reached 40% cumulative release approximately three days earlier than PDGF. Reversing their spatial positions caused PDGF to reach the same threshold about five days earlier than VEGF, while increasing shell thickness further slowed release [83]. These observations show that construct geometry can regulate both transport rate and the sequence of multi-factor presentation.

These studies illustrate two complementary strategies for signal control. Polymer–growth factor affinity regulates molecular retention, whereas printed architecture governs transport distance and release sequence. Integrating these mechanisms may therefore provide more precise temporal matching between signal availability and regenerative demand.

Across Section 5.1, Section 5.2, Section 5.3, Section 5.4 and Section 5.5, the available quantitative evidence shows that bioink properties are strongly coupled rather than independently tunable. Table 5 therefore compares representative systems according to formulation, experimentally varied parameters, quantitative outcomes, and associated trade-offs. Because fabrication protocols and biological models differ among studies, absolute values should not be treated as universal design thresholds. Instead, the within-study changes provide experimentally supported relationships between polymer/network variables and bioink performance. Moreover, fabrication and regenerative requirements do not always align. Increasing the polymer concentration, crosslink density, or gelation rate may improve shape fidelity while restricting cell spreading and matrix remodeling. Thus, the most printable bioink is not necessarily biologically optimal. Bioink design should instead target an application-specific fabrication–regeneration window that maintains sufficient structural fidelity without compromising subsequent tissue maturation. This window is inherently dynamic as mechanical and transport requirements evolve during regeneration.

## 6. Functional Hydrogel Bioinks for Skin Regeneration

**Figure 7:**
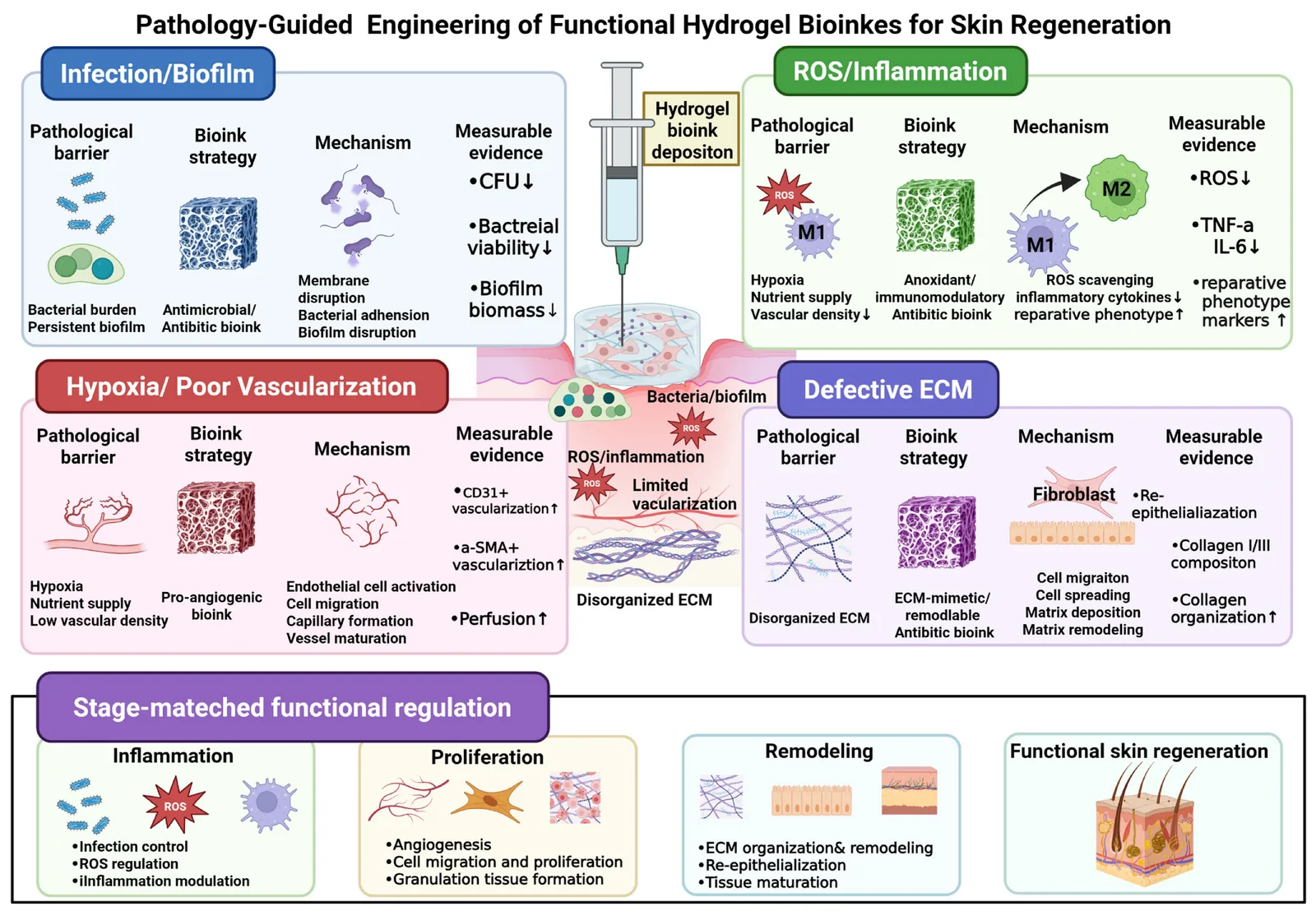
Pathology-Guided Engineering of Functional Hydrogel Bioinks for Skin Regeneration. The figure illustrates four major pathological barriers to wound healing: infection/biofilm formation, oxidative stress and inflammation, hypoxia and insufficient vascularization, and defective extracellular matrix (ECM) remodeling, together with corresponding bioink strategies, mechanisms, and measurable outcomes. The lower panel shows stage-matched functional regulation during inflammation, proliferation, and remodeling to promote functional skin regeneration. Arrows indicate the transition from M1 to M2 macrophage phenotypes, while upward (↑) and downward (↓) arrows indicate increases and decreases, respectively, in the corresponding indicators. Abbreviations: CFU, colony-forming units; ECM, extracellular matrix; ROS, reactive oxygen species; TNF-α, tumor necrosis factor alpha; IL-6, interleukin-6; α-SMA, alpha-smooth muscle actin. Created in BioRender. Zhu, X. (2026) https://BioRender.com/mtimzmn.

The integration of polymer design, network engineering, and functional modification enables hydrogel bioinks to meet the dynamic and multifaceted requirements of skin tissue regeneration. Normal wound healing involves coordinated phases of hemostasis, inflammation, proliferation, and remodeling. However, severe injuries, burns, and chronic wounds such as diabetic ulcers often disrupt this sequence, resulting in persistent inflammation, bacterial infection, impaired vascularization, and delayed tissue repair [84]. To overcome these limitations, functional hydrogel bioinks are increasingly being developed beyond mechanical scaffolds toward instructive microenvironments capable of regulating key biological processes during wound healing. In this section, we discuss how engineered bioinks modulate immune responses, promote angiogenesis, control infection, and facilitate tissue reconstruction in skin regeneration applications. Rather than incorporating multiple functions indiscriminately, functional bioink design should be guided by the primary pathological barriers that limit wound healing. As illustrated in Figure 7, infection, persistent oxidative and inflammatory stress, insufficient vascularization, and defective tissue reconstruction can be translated into distinct functional requirements for polymer networks, thereby enabling pathology-guided and mechanism-matched bioink engineering.

### 6.1. Antimicrobial Bioinks for Infection Control

Bacterial colonization and biofilm formation can sustain inflammation and delay chronic wound repair; therefore, antimicrobial bioinks must achieve effective bacterial suppression without compromising host-cell viability. Antimicrobial activity can be introduced through antibacterial polymers, antibiotics, peptides, or inorganic nanoparticles, while the polymer network regulates their local concentration and release [85].

Antimicrobial efficacy is usually concentration-dependent, so it should be assessed in tandem with cytotoxicity. In GelMA bioinks loaded with superparamagnetic iron oxide nanoparticles (SPIONs), concentrations of 200 and 500 μg/mL reduced S. aureus luminescence by approximately 31% and 45%, respectively. However, fibroblast viability at day 14 was significantly reduced at 500 μg/mL. Ultimately, 200 μg/mL provided the best overall balance between antibacterial activity and cell compatibility [86]. Similarly, silver-loaded GelMA showed a dose-dependent antibacterial effect. Increasing the nanosilver concentration from 50 to 100 mg/L enlarged the inhibition zone against S. aureus from 0.7 to 2.1 mm, while 100 mg/L of nanosilver significantly suppressed fibroblast proliferation during culture [87].

Release kinetics are another critical determinant. One study reported that a ciprofloxacin-loaded gelatin/alginate printed scaffold released approximately 70% of the drug within 5 min and achieved nearly 100% inhibition of E. coli and S. aureus within 24 h [88]. Such strong early antibacterial activity illustrates the benefit of rapid drug availability, but the pronounced burst release also highlights the need to evaluate whether antimicrobial concentrations can be maintained over longer treatment periods. Thus, antimicrobial bioinks should be assessed using combined endpoints of bacterial reduction or biofilm inhibition, release kinetics, and mammalian cell viability rather than antibacterial activity alone.

### 6.2. Immunomodulatory and Redox-Regulating Bioinks

Excessive reactive oxygen species (ROS) and persistent inflammation are major barriers to chronic wound healing, particularly in diabetic wounds. Antioxidant and immunomodulatory bioinks aim to restore local redox homeostasis and regulate inflammatory responses while supporting tissue regeneration [89]. Such regulation can reduce oxidative damage to cells and extracellular matrix components and facilitate subsequent tissue repair.

A representative example is a 3D-printed nanoparticle-enhanced bionic skin (3D-NEBS) that incorporates polydopamine–gallium–arginine nanoparticles into a composite hydrogel composed of oxidized Tremella fuciformis polysaccharide, carboxymethyl chitosan, and fish gelatin. Under 100 μM H_2_O_2_-induced oxidative stress, 3D-NEBS significantly reduced intracellular ROS. Macrophage assays further showed decreased expression of the pro-inflammatory markers iNOS and CD86 and increased expression of the pro-regenerative markers Arg-1 and CD206. TNF-α and IL-6 levels were consistently reduced, whereas IL-10 and TGF-β1 were increased. In diabetic rats, this coordinated regulation of oxidative stress and immune responses resulted in 92.79 ± 0.39% wound closure by day 14 [90].

However, antioxidant activity may decline as reactive groups are consumed, limiting long-term protection in highly oxidative wound environments. Moreover, ROS levels change dynamically across different stages of healing. Future bioink design should therefore emphasize adaptive or sustained redox regulation rather than indiscriminate ROS elimination while maintaining long-term immunomodulatory activity and cellular compatibility.

### 6.3. Angiogenic and Vasculature-Instructive Bioinks

Insufficient vascularization remains a major barrier to the survival and maturation of engineered skin constructs, particularly in thick or metabolically active tissues where diffusion alone cannot sustain long-term oxygen and nutrient supply [4]. Angiogenic bioinks therefore aim not only to stimulate endothelial activity but to also support the formation of organized and stable vascular networks. This requires coordinated regulation of biochemical signaling, endothelial cell migration and organization, and matrix remodeling [91].

A representative example is the heparin-functionalized GH/HepMA bioink developed by Li et al., which consists of 0.3% (*w*/*v*) hyaluronic acid, 10% (*w*/*v*) GelMA, and 0.5% (*w*/*v*) heparin methacrylate. Covalently incorporated heparin enabled sustained VEGF delivery for 21 days, and the VEGF-containing bioink enhanced HUVEC proliferation and migration while also promoting the formation of denser capillary-like networks. When human dermal fibroblasts and HUVECs were spatially incorporated into 3D-bioprinted dermal constructs, the system further supported collagen I/III deposition and the formation of mature capillary-like structures [82].

Moreover, successful angiogenesis should be evaluated beyond the presence of endothelial markers or increased microvessel density. Relevant endpoints should include endothelial migration, network connectivity, lumen formation, vascular maturation, and, ultimately, functional perfusion and integration with host vessels. In particular, the GH/HepMA study primarily demonstrated prevascularization in vitro. Whether these capillary-like networks rapidly anastomose with host vasculature and provide functional blood flow in vivo remains to be established. Vasculature-instructive bioink design should therefore integrate controlled angiogenic signaling with matrix remodeling and spatial cellular organization to promote both vascular formation and functional maturation.

### 6.4. ECM-Mimetic and Regenerative Bioinks for Tissue Reconstruction

ECM-mimetic and regenerative bioinks are designed to recreate key structural and biochemical features of the native skin ECM and thereby support coordinated tissue reconstruction. Rather than simply accelerating wound closure, regenerative bioinks should promote reconstruction of the epidermal–dermal architecture and progressive replacement of the printed matrix by newly formed tissue. To construct full-thickness human skin equivalents, Yang et al. incorporated recombinant human type III collagen (rhCol3), a major structural component of native skin ECM, into GelMA. The optimized bioink contained 7.5 wt% GelMA and up to 3.2 wt% rhCol3 and maintained approximately 90% cell viability after extrusion bioprinting. Human dermal fibroblasts were encapsulated within the printed dermal layer, followed by seeding of keratinocytes to reconstruct the epidermis. Increasing the rhCol3 content promoted early keratinocyte expansion, producing near-complete surface confluence by day 3. The resulting constructs yielded an epidermal layer of approximately 40 μm. In a full-thickness rat wound model, the GelMA–rhCol3-3.2 formulation achieved approximately 94% wound closure by day 14, significantly exceeding GelMA alone and untreated controls; histological analysis further revealed new collagen deposition and newly formed hair follicles in the regenerated dermis [92]. These findings show that ECM-mimetic bioinks cannot be assessed by cell viability or wound-closure rate alone. Tissue quality also matters, including epidermal stratification, ECM deposition and organization, dermal remodeling, and the restoration of skin appendages. Nevertheless, in current models, regenerated tissue still falls short of native skin in its full architectural and functional complexity. Future bioinks should therefore combine tissue-specific ECM signals with controllable degradation and spatial organization to facilitate progressive matrix replacement and more complete functional skin regeneration.

## 7. New Biofabrication Strategies

Extrusion-based bioprinting remains the dominant method for fabricating hydrogel constructs, but limitations in printing speed, spatial resolution, and architectural complexity constrain its application in advanced skin regeneration. Emerging biofabrication strategies aim to overcome these limitations by combining engineered polymer networks with more precise and adaptable manufacturing approaches. These technologies expand the ability to control construct geometry, fabrication efficiency, and biological organization, thereby broadening the design space for skin-regenerative bioinks.

### 7.1. Multi-Material and Coaxial Bioprinting for Spatially Heterogeneous Constructs

Native skin is spatially heterogeneous, with distinct tissue compartments differing in their cellular composition, matrix organization, and mechanical properties. Multi-material bioprinting addresses this complexity by spatially organizing different bioinks or cell populations within a single construct, enabling compartment-specific design^181^. Coaxial bioprinting extends this strategy by generating core–shell or hollow filaments through concentric extrusion. Such architectures are particularly useful for vascular biofabrication because perfusable or endothelialized channels can be formed directly during printing [8]. This configuration is particularly advantageous for vascular biofabrication because hollow or endothelialized channels can be generated directly during printing. For example, Jia et al. used a multilayer coaxial nozzle together with a GelMA/alginate/PEGTA bioink to directly fabricate perfusable vascular constructs in a single printing process [93]. More recently, Wang et al. developed a one-step coaxial strategy for pre-vascularized skin organoids using an endothelial cell-containing sacrificial core surrounded by a biomimetic hydrogel containing fibroblasts and adipose-derived stem-cell microspheres [94]. The main challenge is maintaining compatibility between adjacent bioinks and stable control of the core–shell interface. Differences in rheology or crosslinking behavior can disrupt filament geometry and structural integration, while increased nozzle complexity may introduce additional processing variability. Multi-material design should therefore prioritize interfacial compatibility and spatial control rather than material diversity alone.

### 7.2. Light-Based Bioprinting: Digital Light Processing (Dlp) and Volumetric Fabrication

DLP and volumetric bioprinting provide rapid, high-resolution alternatives to filament-based extrusion by using spatially controlled photopolymerization [95]. This capability is particularly relevant to skin biofabrication, where vascular features and tissue interfaces require precise spatial organization. DLP-compatible bioinks typically rely on photocrosslinkable polymers, such as GelMA, PEGDA, or HAMA, together with cytocompatible photoinitiators.

DLP selectively polymerizes predefined regions using patterned light, whereas volumetric bioprinting reconstructs three-dimensional structures through tomographic projection without sequential layer deposition [96]. The latter can substantially shorten fabrication time and reduce layer-dependent defects. Light-based systems have consequently been used to generate cell-laden constructs and microscale vascular architectures with high structural fidelity [97].

Their main limitations arise from the optical and material requirements of photopolymerization. Light attenuation and scattering can reduce curing uniformity in cell-dense or compositionally complex bioinks, while low-viscosity precursors may provide insufficient structural stability before crosslinking. Future development should therefore focus on photoresponsive polymer systems that combine rapid curing, adequate mechanical support, and reliable light penetration rather than pursuing resolution alone.

### 7.3. In Situ Bioprinting for Direct Wound Management

In situ bioprinting represents a clinically oriented biofabrication strategy that enables direct deposition of cell-laden or bioactive polymer inks onto anatomical defects without the need for prefabricated scaffolds or extensive ex vivo maturation [98]. This approach is particularly attractive for irregular skin wounds because material deposition can be adapted directly to patient-specific wound geometry. Suitable bioinks therefore require rapid stabilization, cytocompatibility, tissue conformity, and sufficient adhesion under physiological conditions.

Integration with handheld printing, optical scanning, or robotic positioning can further improve spatial control and adaptation to complex wound contours. Direct deposition may enhance wound coverage and tissue–material contact while enabling the localized delivery of regenerative components [99,100]. Recent studies have demonstrated that handheld bioprinting of cell-laden GelMA- or HA-based bioinks for skin repair showed enhanced re-epithelialization and improved tissue organization in large-animal wound models [101]. Despite its translational potential, in situ bioprinting faces substantial challenges, including maintaining sterility in clinical environments, compensating for patient movement during deposition, ensuring uniform crosslinking under physiological conditions, and minimizing potential toxicity associated with photoinitiators or reactive intermediates. Future development should therefore prioritize robust wound-compatible bioinks and accurate delivery systems capable of translating patient-specific wound geometry into reproducible regenerative constructs.

### 7.4. Four-Dimensional Bioprinting and Shape-Programmable Hydrogel Networks

Unlike the molecular-scale relaxation and remodeling discussed in Section 3.4, 4D bioprinting deliberately encodes post-fabrication changes in macroscopic shape or function. By combining stimuli-responsive polymers with spatially programmed network architectures, printed constructs can undergo controlled transformations after fabrication in response to environmental cues [12]. A key advantage of this approach is that relatively simple printed geometries can subsequently transform into more complex structures. For example, shape-morphing alginate- and hyaluronic acid-based hydrogels have been designed to spontaneously form hollow tubular structures with dimensions approaching those of small blood vessels [102]. Shape-programmable hydrogels have also been explored for minimally invasive deployment, while thermoresponsive 4D-printed dressings have demonstrated improved conformity to irregular wounds and temperature-triggered contraction [103]. These strategies suggest that programmed shape changes could improve wound conformity and generate spatially organized structures without requiring the final geometry to be produced entirely during printing. Despite this potential, the application of 4D bioprinting in skin regeneration remains at an early stage. Transformation behavior is sensitive to local physiological conditions and mechanical constraints, and excessive or poorly controlled deformation may compromise cell viability or structural integrity. Future development therefore requires predictable transformation kinetics, cytocompatible actuation mechanisms, and precise coordination between programmed structural changes and the evolving biological requirements of wound healing.

### 7.5. AI-Assisted Process Control and Automated Biofabrication

Artificial intelligence (AI) and automated biofabrication are increasingly used to streamline process optimization, structural design, and manufacturing control in bioprinting processes. Machine learning models can relate the properties of bioinks and printing conditions to structural outcomes, helping identify suitable processing windows and reduce empirical trial and error [104]. Computational and image-based tools can further support patient-specific design and fabrication planning.

More importantly, automation extends the use of AI from pre-printing optimization to real-time process control. Computer vision and process sensing can detect printing deviations, while data-driven algorithms can adjust fabrication parameters during deposition. When coupled with robotic systems, these functions provide a basis for closed-loop biofabrication in which sensing, prediction, and corrective control are continuously integrated. For skin regeneration, such systems may improve reproducibility and facilitate adaptation to patient-specific wound geometries and multilayered constructs [105].

However, most AI-assisted biofabrication systems remain dependent on small, laboratory-specific datasets, limiting transferability across printers, bioinks, and experimental conditions. Standardized datasets, external validation, and robust cross-platform performance are therefore required for these systems to support reliable manufacturing. The principal value of AI is not in replacing biofabrication expertise but in making manufacturing processes more reproducible, adaptive, and controllable [106].

## 8. Obstacles to Clinical Translation

Despite substantial advances in polymer design, functional bioink development, and emerging biofabrication technologies, the clinical translation of 3D-bioprinted skin constructs remains limited. Existing research has largely demonstrated proof-of-concept regeneration in controlled laboratory or preclinical models. In reality, translating these innovations into clinically reliable therapies requires overcoming complex challenges spanning biological performance, manufacturing reproducibility, scalability, and regulatory approval. Unlike conventional biomaterials, bioprinted skin constructs require the coordinated integration of polymers, living cells, bioactive factors, and dynamic fabrication processes, creating additional requirements for safety, quality control, and long-term functional validation.

### 8.1. Biological Complexity and Functional Reconstruction

Although progress has been made regarding the application of 3D-bioprinted skin constructs in wound closure and tissue repair, reproducing the full biological complexity and functional properties of native skin remains a major challenge. Native skin is a highly organized tissue composed of multiple interacting components, including epidermal and dermal layers, vascular networks, immune cells, neural structures, and appendages. Current biofabrication strategies can partially reconstruct tissue architecture but are still insufficient to achieve fully functional skin regeneration [107]. A major limitation is the establishment of mature, perfused vasculature that integrates effectively with host circulation. Although bioprinted vascular channels and endothelialized structures can improve cell survival, achieving stable vascular perfusion remains difficult [108]. Similarly, engineered constructs must support coordinated immune responses that enable early defense, inflammation resolution, and tissue remodeling. The reconstruction of hair follicles, sweat glands, pigmentation units, and sensory nerves also remains challenging because these structures require coordinated developmental signals and precise spatial organization [109]. These limitations indicate that clinical success should be evaluated according to factors other than wound closure alone. In addition, future development should prioritize durable functional recovery over structural resemblance alone.

### 8.2. Bioink Standardization and Critical Quality Attributes

The lack of standardized formulations and manufacturing protocols remains a major barrier to the clinical translation of hydrogel bioinks. Many existing bioinks rely on natural polymers whose composition, molecular weight, and biological activity can vary substantially between batches. Such variability may alter network formation and subsequently affect printability, degradation behavior, and biological performance [110]. Clinical development therefore requires qualified raw materials and Good Manufacturing Practices (GMP)-compatible manufacturing processes with validated controls over material preparation, crosslinking, and cell incorporation. Quality control should extend beyond the precursor bioink to the final construct. Product-specific critical quality attributes (CQAs) should be defined according to the intended clinical function, with acceptance criteria established for the final construct. For cell-laden products, release testing should also confirm that manufacturing preserves cellular function. Consistent acceptance criteria and validated analytical methods are essential to ensuring batch-to-batch reproducibility and product consistency during scale-up. Sterility represents an additional challenge because terminal sterilization is often unsuitable for constructs containing living cells. Sterility assurance therefore depends on appropriate raw-material treatment, validated aseptic processing, and suitable microbiological testing. Sterilization procedures for polymer components should also be evaluated for their effects on polymer chemistry and network formation. Sterility and product quality must consequently be validated together. Future standardization should integrate raw-material control, process validation, and product-specific release criteria to support consistent and clinically suitable bioink manufacturing.

### 8.3. Cell Source and Biological Safety

Although the use of living cells enables 3D-bioprinted skin constructs to actively support tissue regeneration, it introduces challenges in cell sourcing and long-term biological safety [111]. Existing strategies mainly employ primary skin cells, mesenchymal stem cells (MSCs), induced pluripotent stem cell (iPSC)-derived cells, or cell-free approaches, each with distinct advantages and limitations. Autologous keratinocytes and fibroblasts offer favorable immune compatibility but require invasive harvesting and prolonged cell expansion, whereas allogeneic and stem cell-derived populations may improve availability while introducing additional safety concerns. In particular, stem cell-based constructs require careful evaluation of genomic stability, unwanted differentiation, and potential abnormal proliferation before clinical application [112].

Because cell-related adverse effects may emerge after implantation, long-term follow-up should assess phenotypic stability, host immune responses, and delayed abnormal tissue changes. These concerns are particularly relevant in complex wounds, where persistent inflammation and infection may affect the behavior of implanted cells and their interactions with the host tissue [13]. Clinical translation therefore requires standardized cell sourcing and validated safety assessments that establish not only short-term cell viability but also stable and predictable biological behavior after implantation.

### 8.4. GMP-Compatible Manufacturing, Process Control, and Reproducibility

Clinical translation requires transitioning from laboratory-scale bioprinting to a controlled manufacturing process that can consistently reproduce the intended product. Manufacturing requirements should be aligned with the construct’s regulatory classification and intended use. GMP-compatible manufacturing therefore requires predefined material specifications, validated production procedures, and documented process controls rather than simply scaling up an experimental printing protocol [113]. Reproducibility remains challenging because bioprinting combines material processing with living biological components. Variations in bioink handling or printing conditions can alter construct architecture and cellular performance, particularly as construct size and complexity increase. Automated fabrication and in-process monitoring can help reduce operator-dependent variability and detect manufacturing deviations during printing. Rather than relying exclusively on endpoint testing, monitoring during printing can identify deviations before they affect the final construct. Upstream processes, including the preparation of cells and bioink components, must also remain consistent with downstream maturation and product-release requirements.

Batch-to-batch reproducibility must be demonstrated by evaluating the final constructs, rather than inferred from identical printing settings alone, when changes to raw materials, equipment, or manufacturing procedures are needed to determine whether product quality and function are maintained. The goal of clinical-scale manufacturing is therefore not simply to increase throughput but to establish a reproducible process capable of maintaining good quality throughout a product’s lifecycle.

### 8.5. Regulatory Classification and Clinical Validation

Regulatory classification depends on product composition, intended use, and primary mode of action. Acellular constructs may follow medical-device pathways, whereas constructs containing viable cells may be regulated under biological or tissue-engineered product frameworks. Products combining cellular and device-like components may require combination-product regulation. In the United States, the lead regulatory center for a combination product is determined by its primary mode of action, whereas in the European Union, engineered cell- or tissue-containing products intended to regenerate, repair, or replace human tissue may fall under the advanced therapy medicinal product (ATMP) framework.

Clinical validation is a related challenge. Current preclinical models cannot fully reproduce the heterogeneity of human wounds, particularly chronic wounds associated with systemic diseases [114]. Preclinical evaluation should therefore reflect the intended clinical indication and generate evidence relevant to subsequent clinical testing. FDA guidance on chronic cutaneous ulcers and burn wounds identifies complete wound closure as an important clinical endpoint. However, durable closure and longer-term follow-up are also needed to determine whether regenerated tissue remains stable and restores clinically relevant function.

From a translational perspective, we argue that the major bottleneck may no longer be the lack of increasingly sophisticated polymer systems but rather the insufficient control of formulation complexity, manufacturing reproducibility, and product quality. Many multifunctional bioinks perform well under laboratory conditions, yet batch-to-batch variation, sterilization compatibility, storage stability, scale-up behavior, and critical quality attributes remain far less systematically studied. Each added polymer component can improve functionality while increasing manufacturing uncertainty and regulatory complexity. Highly multifunctional formulations should therefore not be regarded as automatically superior for clinical translation. In some cases, chemically well-defined, relatively simple polymer systems with controllable manufacturing processes may ultimately offer greater translational value than elaborate formulations that yield only marginal biological benefit at the expense of reproducibility.

Successful clinical implementation also depends on the feasibility of incorporating the product into routine treatment workflows. Regulatory planning, manufacturing control, and clinical validation should therefore be integrated into material development from the outset rather than addressed only after bioink optimization.

## 9. Future Perspectives

The rapid evolution of polymer chemistry, biofabrication, and regenerative medicine has transformed hydrogel bioinks from passive matrices into increasingly instructive material systems, yet their design remains largely empirical, and the relationships between polymer structure, network behavior, and in vivo regeneration are still insufficiently predictive. Future research should shift toward quantitative and data-guided polymer engineering capable of linking material design with biological performance and translational requirements.

### 9.1. From Empirical Formulation to Predictive Polymer Design

Current bioink development still relies heavily on iterative formulation and process optimization. Although effective, this trial-and-error approach is inefficient and provides limited ability to predict how changes in polymer design will propagate through network behavior to fabrication and biological outcomes. Future development should therefore move toward predictive and inverse design, in which the desired material or regenerative performance is defined first and suitable polymer formulations are subsequently identified. Data-driven modeling could support this transition by linking polymer and network characteristics with experimentally measured performance. However, its predictive value depends on datasets that are sufficiently standardized and comparable across studies. Differences in characterization methods, printing protocols, and biological models currently limit model transferability and reduce the reliability of cross-study prediction. Progress will therefore require closer integration of standardized experimental data, mechanistic understanding, and computational modeling. Such integration could reduce empirical screening and enable more rational, application-specific design of polymer bioinks.

### 9.2. From Responsive Networks to Closed-Loop Adaptive Systems

Skin regeneration occurs within a continuously changing microenvironment, whereas most current responsive bioinks are designed to react to predefined stimuli. Future polymer systems should therefore progress toward closed-loop platforms that can sense changes in the wound environment and adjust their behavior accordingly. Rather than delivering a fixed response, such systems could dynamically regulate material or therapeutic functions as healing progresses. Integrating real-time sensing with adaptive material responses could enable bioinks to respond more precisely to the evolving regenerative state. The key advance is therefore not simply greater stimulus responsiveness but the development of coordinated sense–respond–adapt systems. Achieving this transition will require reliable sensing, predictable network responses, and close coupling between material adaptation and biological healing processes.

### 9.3. From Cell-Laden Bioinks to Engineered Living Systems

Conventional cell-laden bioinks primarily use cells as regenerative components embedded within polymer matrices^208^. Future systems may extend this concept by integrating engineered cells with functional polymer networks to create living platforms capable of actively modulating the wound microenvironment. Rather than serving only as tissue-forming elements, engineered cells could be programmed to sense local conditions and produce regenerative signals as healing progresses while the surrounding polymer network provides structural and spatial control. This shift would transform cells from passive biological cargo into programmable regulators of regeneration.

Still, the transition toward engineered living systems requires stringent control over cellular behavior and long-term function to ensure predictable and safe performance. Future development should therefore balance biological autonomy with external controllability, allowing engineered living bioinks to remain responsive without sacrificing reproducibility or translational feasibility.

### 9.4. From Personalized Bioinks to Clinically Deployable Platforms

Future bioink development is expected to move from universal formulations toward patient-specific regenerative platforms. Because wound conditions vary substantially among patients, it may be necessary to adapt material design to the biological and structural requirements of individual lesions. Integrating patient- and wound-specific information with computational material design could guide the selection of suitable polymer properties and fabrication strategies. Meanwhile, this personalization must remain compatible with reproducible manufacturing and clearly defined quality requirements. The ultimate goal is therefore not unrestricted customization but clinically practical personalization within a controlled manufacturing framework.

Increasing biological complexity should not in itself be regarded as the ultimate objective of bioink design. Although increasingly biomimetic systems may enhance functionality, additional components also introduce greater variability and complicate manufacturing and regulatory control. We therefore propose a principle of minimum sufficient complexity: a bioink should incorporate only those material and biological functions necessary for the intended regenerative outcome. Inverse design should begin with the required tissue function and clinical constraints, followed by identifying the simplest polymer and network features capable of meeting those needs.

## 10. Conclusions

Hydrogel bioinks lie at the intersection of polymer science, biofabrication, and regenerative medicine. Their performance is governed by multiscale relationships linking polymer molecular design and network architecture to material behavior and regenerative function. Bioink development should therefore move beyond empirical material selection toward rational control of structure–property–function relationships.

For skin regeneration, suitable polymers should balance reliable fabrication with a microenvironment that remains sufficiently stable yet permissive to cellular remodeling. No single polymer fully satisfies these requirements: GelMA combines cell-adhesive functionality with tunable photocrosslinking and alginate offers favorable printability but limited intrinsic bioactivity, whereas hyaluronic acid is highly relevant to the skin extracellular environment but often requires structural reinforcement. These complementary strengths and limitations suggest that optimal bioinks should be designed by matching polymer chemistry and network structure to regenerative and manufacturing needs rather than by maximizing individual material properties.

Future progress will depend on establishing quantitative relationships between polymer design, network behavior, and biological outcomes while maintaining reproducible and clinically compatible manufacturing methods. The ultimate goal is therefore not simply a highly printable or multifunctional bioink but a predictable and controllable polymer system capable of supporting durable skin regeneration and clinical translation.

## Figures and Tables

**Table 1:** Top 15 journals by total citations in the bibliometric dataset, together with publication output and H-index.

Rank	Sources	Articles	TC	H-Index
1	Nature Biotechnology	1	5463	1
2	Biomaterials	10	3260	9
3	International Journal of Biological Macromolecules	61	2192	27
4	Pharmaceutics	11	2164	10
5	Acta Biomaterialia	19	1853	13
6	Bioactive Materials	20	1625	15
7	Advanced Healthcare Materials	27	1459	15
8	Biotechnology Advances	1	1364	1
9	Polymers	25	1328	16
10	Biomacromolecules	7	1297	5
11	Biomaterials Science	20	1170	16
12	International Journal of Molecular Sciences	18	1072	14
13	International Journal of Bioprinting	36	1041	15
14	Biofabrication	10	932	7
15	Acs Applied Materials & Interfaces	10	902	9

**Table 2:** Top 10 most globally cited publications in the bibliometric dataset.

Rank	First Author	Title	Year	Journal IF	TC	TCpY
1	Murphy SV [15]	3D bioprinting of tissues and organs	2014	Nat. Biotechnol.	5463	420.2
2	Mandrycky C [16]	3D bioprinting for engineering complex tissues	2016	Biotechnol. Adv.	1364	124
3	Tottoli EM [17]	Skin Wound Healing Process and New Emerging Technologies for Skin Wound Care and Regeneration	2020	Pharmaceutics	1240	177.1
4	Matai I [18]	Progress in 3D bioprinting technology for tissue/organ regenerative engineering	2020	Biomaterials	918	131.1
5	Klotz BJ [19]	Gelatin-Methacryloyl Hydrogels: Towards Biofabrication-Based Tissue Repair	2016	Trends Biotechnol.	813	73.9
6	Zhao X [20]	Photocrosslinkable Gelatin Hydrogel for Epidermal Tissue Engineering	2016	Adv. Healthc. Mater.	775	70.5
7	Gudapati H [21]	A comprehensive review on droplet-based bioprinting: Past, present and future	2016	Biomaterials	687	62.5
8	Cui X [22]	Direct Human Cartilage Repair Using Three-Dimensional Bioprinting Technology	2012	Tissue Eng. Part A	629	41.9
9	Xu J [23]	Hydrogels Based on Schiff Base Linkages for Biomedical Applications	2019	Molecules	549	68.6
10	SUN W [24]	Silk Fibroin as a Functional Biomaterial for Tissue Engineering	2021	INT. J. MOL. SCI.	532	88.7

TC, total citations; TCpY, total citations per year.

**Table 3:** Representative quantitative experimental evidence linking polymer molecular and network design parameters to material properties, biofabrication performance, and biological responses.

Design Parameter/Reference	Experimental System	Key Quantitative Findings	Design Implication
Polymer concentration [20]	GelMA, 5–20% (*w*/*v*); methacrylation ≈75%	Compressive modulus: 3 ± 1 → 108 ± 8 kPa; swelling: 1476 ± 28% → 470 ± 9%. Degradation increased from complete degradation within 3 d at 5% GelMA to approximately 20% residual hydrogel after 56 d at 20%. HaCaT viability remained >90%.	Higher concentration enhances stiffness and persistence but reduces swelling; optimize concentration according to tissue-specific requirements.
Degree of functionalization [30]	GelMA with methacrylation degrees of 49.8%, 63.8%, and 73.2%	Compressive modulus: 2.0 → 3.2 → 4.5 kPa; pore size: 49.7 → 30.13 → 23.6 μm. Microvessel density decreased from 58.34 ± 32.70 to 7.20 ± 9.15 vessels mm^−2^ between low- and high-methacrylation groups.	Greater functionalization strengthens the network but may restrict cellular remodeling and vascularization.
Mesh size [33]	PAAm substrates with 6%, 9%, and 12% acrylamide; apparent stiffness maintained at ≈13 kPa	Smaller mesh size significantly increased hBMSC spreading and migration (*p* < 0.0001), focal adhesion formation (*p* < 0.01), and nuclear YAP localization (*p* < 0.0001).	Mesh size regulates cell behavior independently of apparent stiffness and should be treated as a distinct design variable.
Matrix stiffness [34]	5% GelMA/PEGDA; PEGDA increased from 2.5% to 10% (*w*/*v*)	Compressive modulus: 1.3 ± 0.5 → 23.0 ± 1.0 kPa. Fibroblast spreading increased from 920 ± 211 to 2006 ± 188 μm^2^; endothelial sprout length peaked at 30,120 ± 1225 μm at 5 kPa and declined at higher stiffness.	Mechanical reinforcement produces cell-specific, non-monotonic responses; identify an application-specific stiffness window.
Dynamic covalent crosslinking [36]	Hydrazone-crosslinked HELP bioink; small-molecule competitor modulated bond exchange	Baseline storage modulus ≈10^3^ Pa; competitor reduced modulus by up to two orders of magnitude. At 20 mM, gelation occurred within 30 min; at 10 mM, modulus recovered after 24 h in PBS.	Tune bond-exchange kinetics to balance extrusion, gelation, and post-printing recovery.
Interpenetrating polymer network (IPN) [41]	Gel-HDA/GelMA; component ratio varied	Sol–gel transition: 27 → 30 °C; compressive modulus tunable from 20.6 ± 2.48 to 116.7 ± 14.80 kPa. Encapsulated cells proliferated over 10 d.	Complementary polymer phases expand the processing and mechanical design window.
Secondary-network reinforcement [43]	Dynamic-covalent hydrogel reinforced by a photocrosslinkable HA network	Photostiffening increased modulus by approximately 300% and reduced scaffold erosion by approximately 70%.	Sequential reinforcement separates printing-stage processability from post-printing stability.
Nanocomposite reinforcement [45]	NICE bioink: GelMA, κ-carrageenan, and nanosilicates	Compressive modulus: 16.5 ± 1.5 → 71.1 ± 4.9 kPa; storage modulus: 3.1 ± 0.3 → 14.2 ± 1.4 kPa. Post-extrusion cell viability remained >85%.	Polymer–particle interactions reinforce network mechanics without relying solely on increased covalent crosslinking.
Stress-relaxation kinetics [46]	Alginate hydrogels; molecular weight reduced from 280 to 35 kDa, with PEG spacers used to accelerate relaxation	Stress-relaxation half-time decreased from approximately 1 h to 1 min, while initial modulus was maintained. Faster relaxation significantly increased fibroblast spreading and proliferation (*p* < 0.0001).	Network relaxation kinetics regulate cellular remodeling independently of initial stiffness.
Degradation kinetics [47]	PEG-4MAL with 100%, 50%, or 0% MMP-degradable crosslinks	From day 1 to day 7, (G′) decreased by 64% in fully degradable gels and 50% in partially degradable gels. Sprout length reached 961 ± 132 μm and 789 ± 55 μm, respectively, versus 0 μm in non-degradable gels.	Controlled degradation creates space for cellular outgrowth and regulates regenerative signaling; degradation should be coordinated with tissue formation.

Abbreviations: GelMA, gelatin methacryloyl; PAAm, polyacrylamide; PEGDA, poly (ethylene glycol) diacrylate; HELP, hyaluronan–elastin-like protein; Gel-HDA, gelatin–hyaluronan dialdehyde; HA, hyaluronic acid; NICE, nanoengineered ionic–covalent entanglement; PEG-4MAL, four-arm maleimide-functionalized poly (ethylene glycol); MMP, matrix metalloproteinase; hBMSC, human bone-marrow-derived mesenchymal stromal cell; G′, storage modulus.

**Table 4:** Representative polymer platforms for hydrogel bioinks: molecular characteristics, functional roles, limitations, and engineering strategies.

Polymer Platform	Representative Materials	Dominant Molecular Characteristics	Primary Contribution to Bioink Design	Major Limitations	Typical Engineering Strategies
Gelatin-based proteins	Gelatin [32]; GelMA [49]	Denatured collagen-derived chains containing cell-interactive and enzyme-sensitive sequences; GelMA contains photocrosslinkable methacryloyl groups	Provides cell adhesion and remodeling cues; gelatin offers thermoresponsive behavior, whereas GelMA enables tunable photocrosslinking, network stabilization, and broad biofabrication compatibility	Native gelatin shows poor stability at physiological temperature; highly crosslinked GelMA may reduce mesh accessibility, molecular transport, and cell remodeling	Methacryloyl functionalization; control of degree of substitution and polymer concentration; secondary or complementary network formation
Collagen-based proteins	Collagen [50]	Native ECM protein with cell-binding motifs and self-assembling fibrillar structure	Provides a highly biomimetic environment for cell adhesion, migration, and matrix remodeling	Slow fibrillogenesis, weak mechanical strength, limited shape fidelity, and source-dependent variability	Blending with GelMA, alginate, or synthetic polymers; secondary crosslinking; reinforcement with complementary networks
Fibrin-based proteins	Fibrin [51]	Fibrinogen-derived protein network formed through thrombin-mediated polymerization; constituent of the provisional wound matrix	Supports cell adhesion, migration, and vascularization under mild cell-compatible gelation conditions	Weak mechanical stability and relatively rapid degradation	Combination with mechanically stronger polymers; secondary network reinforcement; control of fibrin formation and degradation
Silk fibroin-based proteins	Silk fibroin [52]	Protein polymer containing β-sheet-forming domains with tunable structural organization	Provides greater mechanical stability and structural reinforcement than many other natural protein hydrogels	Native formulations may exhibit insufficient rheological control or slo*w*/*v*ariable gelation for printing	Enzymatic or chemical crosslinking; regulation of β-sheet formation; blending with more bioactive hydrogel-forming polymers
Alginate-based polysaccharides	Alginate [53]	Anionic polysaccharide composed of mannuronic and guluronic acid units; rapidly crosslinks with divalent ions	Provides rapid, mild gelation, favorable rheological control, extrusion performance, and early shape stabilization	Lacks intrinsic mammalian cell-adhesive motifs and shows limited cell-mediated remodeling	Adhesive peptide functionalization; blending with bioactive polymers; dual ionic/covalent stabilization
Hyaluronic acid-based polysaccharides	HA; modified HA [54,55]	Highly hydrated and negatively charged glycosaminoglycan with abundant hydroxyl and carboxyl groups	Provides ECM-like hydration, molecular transport, and receptor-mediated biological interactions; readily chemically modified	Native HA exhibits low mechanical strength, rapid degradation, and poor shape retention	Methacrylation or other chemical functionalization; combination with GelMA or synthetic polymers; covalent/dynamic network formation
Chitosan-based polysaccharides	Chitosan [56]	Cationic polysaccharide containing reactive amino groups; biodegradable and intrinsically antimicrobial	Introduces charge-mediated molecular interactions and antimicrobial functionality relevant to wound bioinks	Limited standalone mechanical and processing performance; properties depend strongly on molecular weight and degree of deacetylation	Chemical functionalization; blending with complementary polymers; incorporation into multifunctional or responsive systems
Complex ECM-derived platforms	Skin-derived or other dECM [57]	Heterogeneous mixture of structural proteins, glycosaminoglycans, proteoglycans, and tissue-associated molecular signals	Provides tissue-specific biochemical information and a complex ECM-like microenvironment difficult to reproduce with individual polymers	Batch variability, weak rheological/mechanical performance, and strong dependence on tissue source, decellularization, digestion, and sterilization	Combination with GelMA, alginate, HA, or synthetic polymers; secondary crosslinking; standardization of source and processing procedures
PEG-based synthetic polymers	PEG; PEGDA; PEGDMA [58]	Hydrophilic, structurally defined and readily functionalized polymer; available in linear and multi-arm architectures	Enables precise regulation of crosslink density, stiffness, degradation, molecular transport, and network architecture	Intrinsically bioinert with weak protein adsorption, cell adhesion, and cell-mediated remodeling	Incorporation of adhesive peptides, degradable or cell-cleavable linkers, ECM molecules, and natural polymer components
PVA-based synthetic polymers	PVA [59]	Hydrophilic polymer rich in hydroxyl groups; properties depend on molecular weight and degree of hydrolysis	Provides water retention, mechanical integrity, and versatile physical or chemical network formation	Limited intrinsic bioactivity and cellular adhesion; slow degradation and potentially restrictive dense networks	Blending with gelatin, chitosan, HA, or degradable polymers; chemical functionalization; formation of complementary networks
Thermoresponsive block copolymers	Pluronic F127 and related PEO–PPO–PEO polymers [60]	Amphiphilic block copolymers undergoing temperature-dependent micellization and reversible sol–gel transition	Provides excellent flow control and thermoresponsive rheology; useful as printable, rheological, or sacrificial components	Rapid dissolution, weak long-term structural stability, and minimal cell–matrix interaction	Chemical functionalization; secondary stabilization; incorporation into composite systems; use primarily as rheological or sacrificial components
Polyurethane-based platforms	PU [61]	Segmented polymers containing tunable soft and hard domains	Provides adjustable elasticity, toughness, mechanical compliance, and degradation	Limited intrinsic biological activity and relatively complex synthesis/processing	Introduction of hydrophilic or degradable segments; peptide/ECM functionalization; blending with cell-interactive hydrogel polymers
Biodegradable polyester-based platforms	PCL, PLA, PLGA [62]	Hydrophobic aliphatic polyesters with tunable molecular weight, crystallinity, mechanics, and hydrolytic degradation	Provides long-term mechanical reinforcement and structural support	Hydrophobicity, poor intrinsic cell interaction, and generally limited suitability as standalone cell-laden hydrogel bioinks	Use as reinforcing frameworks or supporting phases; surface modification; combination with hydrated and bioactive hydrogel phases
Natural–synthetic hybrid platforms	GelMA/PEG, collagen/PEG, natural polymer/PVA and related combinations [63]	Combine biologically interactive natural macromolecules with chemically programmable synthetic polymers	Partially decouples biological signaling from rheological, mechanical, degradation, and structural requirements	Increased formulation complexity; mismatched swelling, degradation, or crosslinking kinetics may generate heterogeneous networks or unstable interfaces	Functional assignment of individual components; optimization of component ratios and crosslinking sequence; compatible orthogonal or staged network formation

**Table 5:** Quantitative comparison of representative polymer hydrogel bioinks across major performance dimensions.

Performance Dimension	Bioink System	Experimental Variable/Comparator	Experimental Condition/Time Point	Quantitative Results
Rheology and printability	Thermosensitive chitosan [71]	2% (A2) vs. 3% (A3) chitosan	Post-shear measurement at 100 s^−1^; printing at 11 mm s^−1^	Post-shear viscosity: 66 ± 15 vs. 663 ± 95 Pa·s; filament width: 1.2–1.5 vs. 0.57 ± 0.04 mm; A3 became continuous at 10 mm s^−1^ with a width of 0.75 ± 0.07 mm
Rheology and printability	PEO–chitosan–PMMA [72]	Composite bioink vs. pure chitosan	37 °C; extrusion pressure 25–50 kPa	Printable viscosity window: 500–550 Pa·s; cell survival: 92% vs. 67% for pure chitosan
Rheology and printability	Dual physically crosslinked supramolecular bioink [73]	β-glycerophosphate: 0.3 → 1.3 M	Post-print structural assessment	Young’s modulus: ~4 → ~130 kPa; dual-crosslinked constructs retained geometry for ≥21 days
Rheology and printability	GelMA-based formulations [49]	100% vs. 75% vs. 50% GelMA	7.5 mm unsupported span; viability at 24 h	Collapse coefficient: 1.10%, 8.20%, 15.10%; cell viability: 76.1%, 77.3%, 80.8%
Mechanical and viscoelastic performance	Porcine skin-derived dECM [74]	1 → 5 mg mL^−1^ dECM	Rheology and fibroblast infiltration	Storage modulus: ~10 → ~1000 Pa; fibroblast infiltration markedly reduced at 5 mg mL^−1^
Mechanical and viscoelastic performance	Alginate/gelatin [75]	Gelatin: 8 → 12 wt%	Mechanical testing; degradation at day 14	Compressive modulus: 25.6 ± 2.7 → 37.9 ± 3.5 kPa; elongation at break: 57.9 ± 4.2% → 92.1 ± 1.3%; degradation: 62.8 ± 2.9% → 26.4 ± 2.4%; pore size: 128.5 ± 16.6 → 79.4 ± 19.7 μm
Transport, swelling, and degradation	GelMA–HAMA vs. adECM–GelMA–HAMA [76]	Addition of adECM	Swelling at 24 h; collagenase degradation at 72 h	Swelling index: 8.95 ± 0.72 → 11.18 ± 0.45; remaining mass: 37.53 ± 4.52% → 53.06 ± 5.16%; pore diameter: 112.16 ± 8.07 → 131.39 ± 6.88 μm
Transport, swelling, and degradation	Gelatin/alginate [77]	GEL-10-ALG vs. GEL-15-ALG vs. GEL-20-ALG	100–200 mM CaCl_2_; degradation to day 35	Mass loss initiated at ~day 10 for GEL-10-ALG and ~day 17 for GEL-15/20-ALG; at day 35, ~20%, ~60%, and 0% initial mass remained, respectively
Transport, swelling, and degradation	GelMA [78]	5% vs. 10% vs. 15% GelMA	Collagenase degradation at 72 h	Remaining mass: 16 ± 2.0%, 43 ± 0.1%, and 64 ± 1.6%; 5% GelMA completely degraded by day 7
Cell–material interactions	RGD-functionalized alginate [79]	2% vs. 5% alginate	Human dermal fibroblasts; days 5 and 28	Day-5 proliferation: 0.14 ± 0.01 vs. 0.19 ± 0.03 (*p* = 0.001); 5% RGD group: 0.24 ± 0.05 at day 28
Cell–material interactions	Double-crosslinked pectin [80]	Ca^2+^: 6 vs. 10 mM at fixed 2 mM RGD	Stress-relaxation testing and fibroblast culture	Relaxation time: ~470 → ~248 s; elastic modulus: 910.3 ± 156.2 → 1347.2 ± 366.1 Pa; faster-relaxing matrices supported fibroblast spreading within ~24 h
Bioactivity and signal presentation	HA/GelMA/HepMA [82]	Covalently incorporated HepMA	VEGF release	Sustained VEGF release for up to 21 days
Bioactivity and signal presentation	3D-printed core–shell heparin hydrogel [83]	Spatial reversal of VEGF and PDGF	Cumulative growth-factor release	Shell-loaded VEGF reached 40% release ~3 days before core-loaded PDGF; after spatial reversal, PDGF reached 40% ~5 days before VEGF

Abbreviations: adECM, adipose-derived decellularized extracellular matrix; dECM, decellularized extracellular matrix; GelMA, gelatin methacryloyl; HAMA, hyaluronic acid methacrylate; HepMA, methacrylated heparin; PDGF, platelet-derived growth factor; RGD, Arg-Gly-Asp; VEGF, vascular endothelial growth factor.

## Data Availability

The bibliographic records used for the bibliometric analyses in this study were retrieved from the Web of Science Core Collection (WoSCC) and Scopus databases using the search strategy described in the Section 2. The processed datasets generated during the bibliometric analysis are available from the corresponding author upon reasonable request.
